# Synthesis of (FeCoNiCuMn)_3_O_4_ spinel-high entropy oxide and green carbon from agricultural waste for supercapacitor application†

**DOI:** 10.1039/d4ra05204h

**Published:** 2024-10-24

**Authors:** Gobinda Chandra Mohanty, Shubhasikha Das, Anu Verma

**Affiliations:** a School of Nano Science and Technology, Indian Institute of Technology, Kharagpur West Bengal 721302 India gobinda2020@kgpian.iitkgp.ac.in; b School of Environmental Science and Engineering, Indian Institute of Technology Kharagpur West Bengal India

## Abstract

This article highlights (FeCoNiCuMn)_3_O_4_ high-entropy oxide prepared *via* liquid state induction melting techniques for supercapacitor application. Nanostructured high entropy oxides have higher active sites to boost the surface redox process with transition metal cations, such as Fe^2+^, Mn^3+^, Ni^2+^, Co^2+^, and Cu^+^, which helps to improve specific power, long-term cyclic stability, and specific capacitance. Melted and ball-milled HEA Nps were annealed to form the high entropy oxide, which uses a positive electrode for supercapacitor application; this results in the highest specific capacitance of 313 F g^−1^ for a current rate of 5 mV s^−1^ for the optimized 3 M KOH electrolyte. The biochar prepared through the pyrolysis of rice straw biochar shows a maximum specific capacitance of 232 F g^−1^ for 5 mV s^−1^. The fabricated aqueous devices display the highest specific capacitance of 83 F g^−1^ at 2 A g^−1^ with a specific energy of 33.4 W h kg^−1^ at 1700 W kg^−1^.

## Abstract 2

This article highlights (FeCoNiCuMn)_3_O_4_ high-entropy oxide prepared *via* liquid state induction melting techniques for supercapacitor application shows XRD, FTIR, FESEM images respectively.

## Graphical abstract

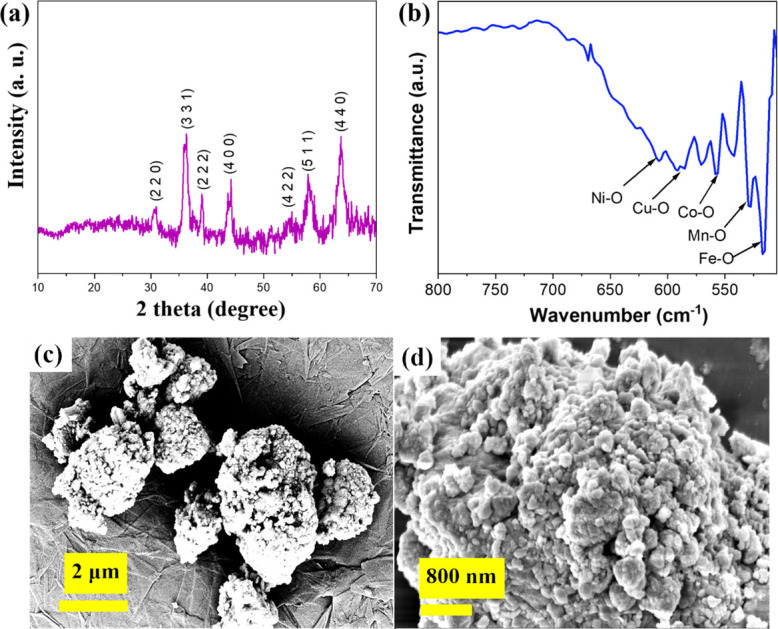

## Introduction

The working characteristics of an electrochemical capacitor are strongly influenced by the properties of the active electrode material used to fabricate the device, the nature of the electrolyte used, and their interaction during electrochemical activities.^1,2^ Further, the fabrication of an asymmetric liquid state device results in higher specific energy with a wide voltage window and long cyclic stability; these excellent characteristics of liquid state devices are due to the high ionic conductivity, electroactivity and high ionic mobility of aqueous electrolytes, which boost electrolytic interaction.^3,4^ Various nanomaterials such as metal oxides, chalcogenides, carbides, and phosphides are used for supercapacitor application.^3^ Recently, there have been studies on high entropy alloys for supercapacitor application. High entropy alloys offer multiple active sites to boost supercapacitive performance.^5^ Moreover, an inductive effect, high entropy effect, and lattice distortion effect result in effective electrolytic ion can participate in electrochemical activities.^6^ Various high entropy alloys are used for energy storage and conversion, energy harvesting, corrosion, and dielectric applications. The supercapacitor field with high entropy alloys has been emerging; however, only few reports are available. Among them, high entropy nanoparticles, high entropy oxides, high entropy alloy-composites as well as high entropy carbides and nitrides have also been reported.^7–9^ In this study, we successfully synthesized (FeCoNiCuMn)_3_O_4_ high entropy oxide through melting, casting and annealing. XRD analysis reveals the spinel structure and FESEM shows the agglomeration morphology of the nanoparticles; furthermore, an in-depth analysis was performed using TEM.

Further oxidation states of quinary elements present in the high entropy oxide were evaluated using XPS analysis. BET and hydrodynamic particle size distribution analyses reveal a specific surface area of 56 m^2^ g^−1^ with an average particle diameter of 400 nm. The prepared electrode shows a maximum specific capacitance of 313 F g^−1^ for a current rate of 5 mV s^−1^ and optimized concentration of potassium hydroxide. In addition, the rice straw biochar prepared by employing pyrolysis techniques shows a maximum specific capacitance of 126 F g^−1^ at 1 A g^−1^. Next, an aqueous asymmetric device was fabricated with the help of FCNCM oxide and biochar positive and negative electrode materials, which shows the highest specific energy of 33.4 W h kg^−1^ at 1700 W kg^−1^. In addition, post-XPS studies show compositional stability even after continuous cyclic studies.

## Materials and method

### Materials

Iron, cobalt, nickel, copper, and manganese were brought from Loba Chemicals India. Potassium hydroxide, carbon black, and graphite sheets are brought from Merck India. *N*-methyl-2-pyroiodine (NMP) and PVDF were purchased from Loba Chemicals India. These chemicals were used as received without further purification.

## Method

### Synthesis of (FeCoNiCuMn)_3_O_4_

The synthesis of (FeCoNiCuMn)_3_O_4_ was done by annealing the Fe–Co–Ni–Cu–Mn HEA powder at 550 °C for 10 h in a tube furnace. Our previous work synthesized Fe–Co–Ni–Cu–Mn HEA powder. An equimolar mixture of the above respective metals is taken for an argon-sealed induction melting at 1350 °C for a series of times to confirm a homogeneous solution; the mixture was melted several times, which was later subjected to a homogenized heat treatment for 48 h at 1000 °C. Next, the solid bulk ingot was parted into several pieces and used for vibratory ball milling to obtain the FeCoNiCuMn HEA powder. The XRD HEA bulk, powder, and oxide analysis confirms a single-phase structure. The XRD pattern reveals a uniform spinel structure of FCC.

### Synthesis of green carbon

The green carbon synthesized from rice straw agricultural waste was mixed with hematite ore (Fe_2_O_3_) in a ratio of 5 : 1. First, rice straw was cleaned with DI water followed by grinding, then mixed with hematite and used for pyrolysis at 600 °C for 1 h. Then, the obtained entity was milled for 2 h to get green carbon, which was analyzed for further characterization and electrochemical studies.

### Characterization

An X-ray diffractometer of D8 Advance Bruker was used for the structural phase analysis. Then, the morphological distribution of HEA oxide was carried out by Zeiss VP-200 along EDAX and elemental mapping. Further, in-depth structural characteristics were obtained with the help of transmission electron microscopy of STEM (JEOL 2100F). In addition, the chemical compositions and oxidation states are evaluated with the help of ThermoFisher Scientific to make Nexsa base. The Raman and FTIR analyses were performed using WITec, UHTS 300 VIS, Germany, and Bruker Alpha II FTIR spectrometer (Bruker Corporation, Germany), respectively.

### Preparation of the electrode

The preparation of the FCNCM oxide electrode follows the ratio 70 : 20 : 10 of the active electrode material, acetylene carbon black, and PVDF; each entity has a significant role in the electrode. The slurry is developed with the help of NMP as the solvent, followed by a mortar pestle grinding and ultrasonication for 30 minutes. Then, the mixture solution is carefully coated into the 1 cm^2^ graphite sheet (after cleaning it with acetone and battery grade). This follows vacuum drying at 70 °C overnight and is used for electrochemical testing. A similar procedure was adopted for the biochar electrode preparation in which the biochar and binder (PVDF) ratio is around 90 : 10. Next, the electrochemical workstation Biologic-E Sp200 was used for the supercapacitor study. Ag/AgCl is used as a reference, and Pt-wire is used as a counter electrode to make the three-electrode measurement system available. The optimized mass loading for the respective electrode for the electrode measurement system is around 1 mg. Biochar and FCNCM oxides are used as negative and positive electrodes to fabricate the asymmetric device for the two electrode measurement systems. A perfect asymmetric combination is approved when a charge-balanced mass loading is applied on both electrodes.

## Results and discussions

**Fig. 1:**
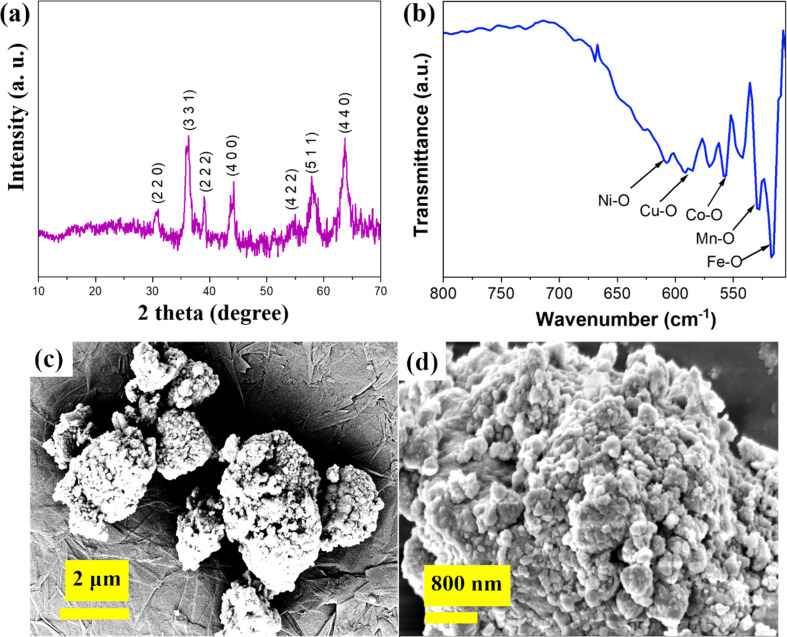
(a) XRD spectrum of (FeCoNiCuMn)_3_O_4_ HEO, (b) FTIR spectrum of HEO, and (c and d) FESEM images of nanostructured FeCoNiCuMn HEO.

**Fig. 2:**
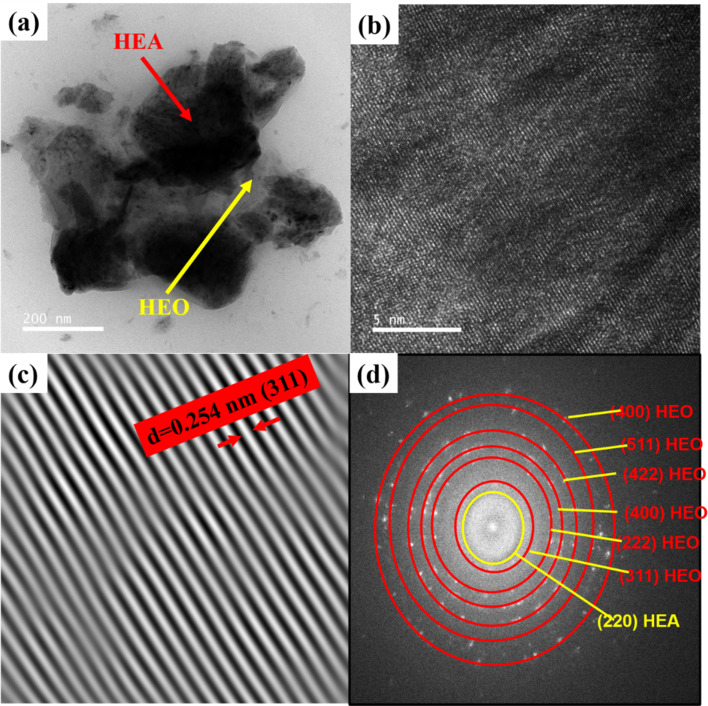
(a) Bright field HRTEM image of (FeCoNiCuMn)_3_O_4_ HEO Nps. (b) STEM image of HEO. (c) Evaluation of lattice spacing by inverse FFT HEO and HEA particles as shown for the HEO with *d* = 0.254 nm for (311) and *d* = 0.287 nm for (220). (d) Corresponding FFT pattern of HEO with identification of each plane.

In the XRD pattern of (FeCoNiCuMn)_3_O_4_ HEO represented in Fig. 1(a), the peaks referring to the spinel type high entropy oxide pattern can be well indexed to cubic FeCr_2_O_4_ (JCPDS No. 34-0140), suggesting the successful synthesis of the single-phase spinel structure (FeCoNiCuMn)_3_O_4_ with the space group of *Fd*3̄*m*. The lattice constant was determined to be *a* = 3.32 Å from the XRD analysis.^10^Fig. 1(b) shows the FTIR spectrum of HEO. Fig. 1(c) and (d) shows the FESEM images of the agglomerated nanostructured HEO. In the lower magnification image Fig. 1(c), the fine particles are in an agglomerated state, and at higher magnification, the fine pores are visible, and clear evidence of a porous structure consisting of (FeCoNiCuMn)_3_O_4_ HEO with opened-up geometry is observed, uniquely displaying a honeycomb analog surface architecture, which is beneficial not only to allow easy penetration of electrolyte ions through its unfold framework but also to ensure good structural stability against rapid charge–discharge cycles. These pores and fine active sites work as epicenters for electrochemical activities. This multivalent quinary high entropy oxide could show good capacitive behavior and uniform morphology distribution. Next, HRTEM analysis was done to get further insight into the details of HEO Fig. 2(a)–(d). As shown in the figure, the bright field image shows the dark area, which is notified as the HEA region and the shadow white region as the HEO region. Further, the FFT pattern selection for evaluating the lattice spacing is around *d* = 0.254 nm (311). Further corresponding FFT pattern of HEO with identification of each plane is shown in Fig. 2(d) respectively.^11^

**Fig. 3:**
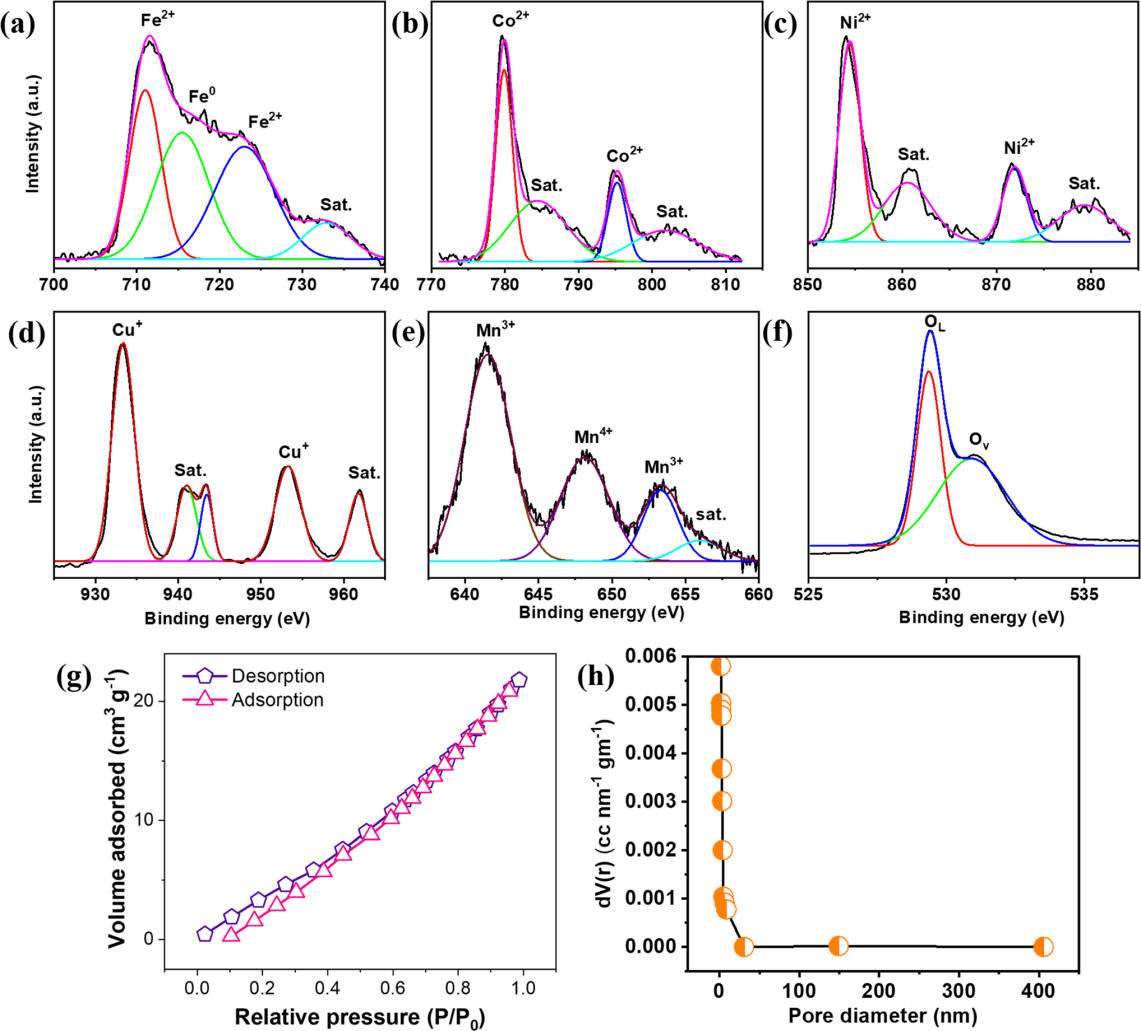
XPS spectrum of (a) Fe 2p, (b)Co 2p, (c) Ni 2p, (d) Cu 2p, (e) Mn 2p and (f) O 1s. (g) BET adsorption/desorption spectrum. (h)Pore size distribution.

Further, XPS studies show multivalent quinary elements with multiple oxidation states, and each element spectrum analyzes the oxidation states in Fig. 3(a)–(f). In addition, the XPS spectrum also shows the presence of all quinary elements. Starting with the Fe 2p spectrum, 711.03 eV (2p_3/2_) and 723 eV (2p_1/2_) refer to Fe^2+^, whereas the peaks at 715.50 eV and 732.92 eV, denoted as satellite peaks, represent the mixed valence state of Fe.^12^ On further analyzing the Co 2p spectrum, 779.87 eV (2p_3/2_) and 795.21 eV(2p_1/2_) refer to Co^2+^, with additional peaks at 784.46 eV and 801.75 eV corresponding to the satellite peaks of Co,^13^. While analyzing the Ni 2p spectrum, 854.43 and 871.89 eV are Ni^2+^ peaks of 2p_3/2_ and 2p_1/2,_ respectively. Furthermore, additional peaks at 860.54 and 879.27 eV are satellite peaks of Ni 2p.^14,15^ Next, for Cu 2p, 933.38 eV and 953.28 eV are referred to as Cu^+^, while other peaks at 941.02, 943.45, and 961.78 eV are mixed valence states of Cu 2p.^16,17^ Analyzing the Mn 2p spectrum, +3 and +4 states of Mn are present; in particular, 641.54, 648.14, 6532.9, and 655.94 eV are referred to as Mn^3+^, Mn^4+^, Mn^3+^, and satellite peaks, respectively.^18,19^ The O 1s spectrum shows peaks at 529.36 eV and 530.91 eV, corresponding to O_L_ and O_V,_ respectively.

The BET spectrum of (FeCoNiCuMn)_3_O_4_ HEO shows adsorption, and the desorption spectrum follows the type-IV trait, as shown in Fig. 3(g). The specific surface area is calculated to be 53 m^2^ g^−1^, which refers to a high specific surface area resulting in higher specific capacitance. In addition, pore size distribution corresponds to most of the pores belonging to the mesoporous region (<50 nm), and these fine pore distributions over the HEO surface benefit higher specific capacitance, as shown in Fig. 3(h). AFM data is added in the Supplementary section, Fig. S9.†

### Characterizations of RS-biochar

#### XRD

**Fig. 4:**
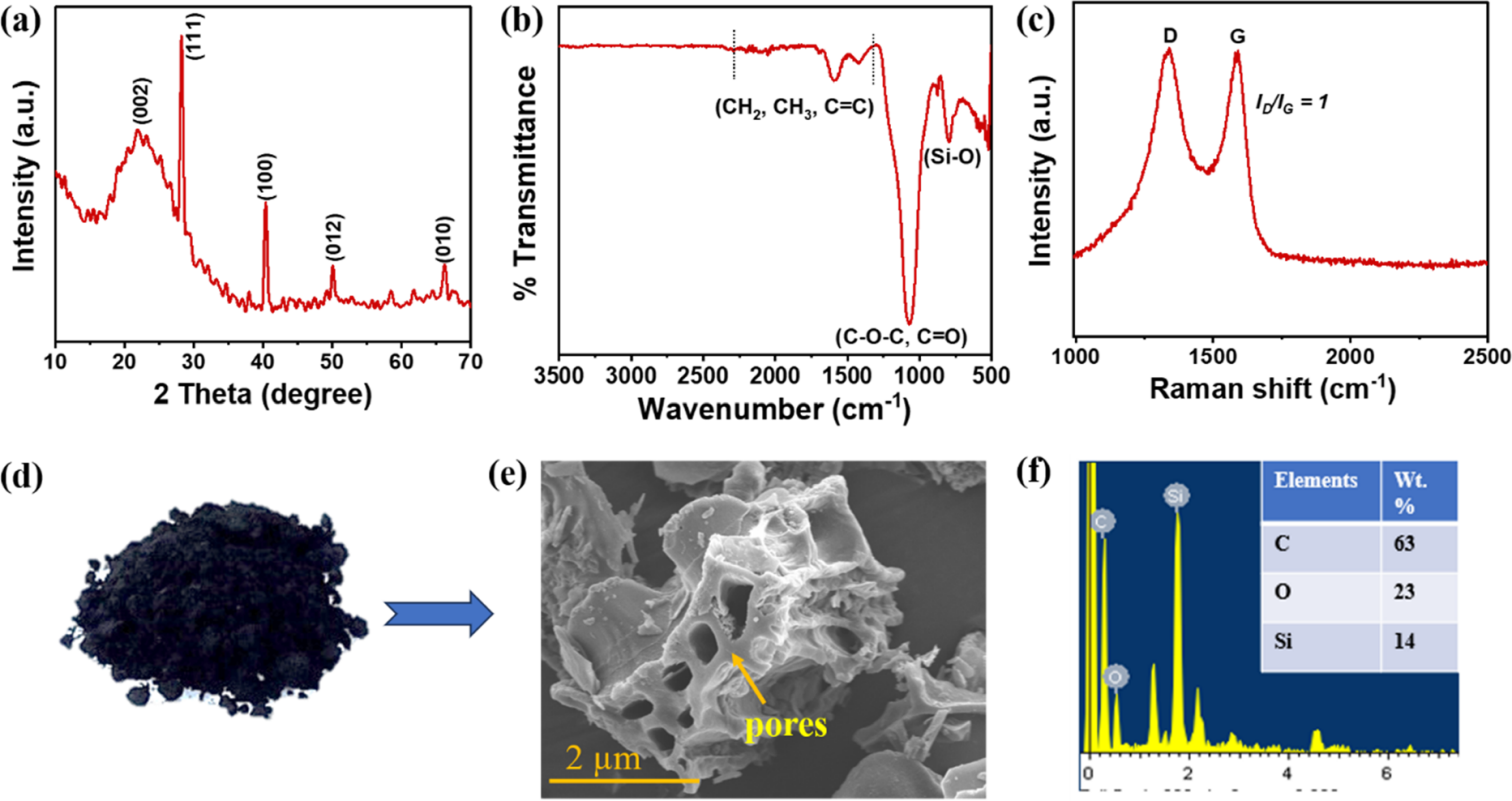
(a) XRD, (b) FTIR, (c) Raman spectra, (d) RSB (biochar), (e) SEM image and (f) EDX analysis.

In the X-the ray diffraction pattern, sharp peaks at 22.1°, 28.9°, and 40° reveal the presence of amorphous carbon (002) and inorganic substances, including SiO_2,_ as shown in Fig. 4(a). The high levels of SiO_2_ in SRB were observed due to low lignin content. The peak located at 22.1° corresponds to the graphite (002) bands, according to ref. 20 and 21. The findings from FTIR spectra also demonstrated that the transition products generated in this temperature range were compatible with this. These tiny bundles of graphene-like analogs are still structured in turbostratic disorder and are consequently known as turbostratic crystallites. In conclusion, the RSB XRD band patterns are composed of an amorphous material with a weak crystalline phase.

#### FTIR

Fig. 4(b) displays the RSB FT-IR spectra. According to ref. 22, the stretching of C–O and C–O–C bonds revealed the peaks at 1080 cm^−1^. For RSB, various peaks (CH_2_, CH_3_, and C=C) between 1440 and 1300 cm^−1^ were observed, derived from cellulose, hemicellulose, and lignin.^23^ The peaks between 1440 and 1300 cm^−1^ refer to a more active aromatic structure developed due to the breakdown of cellulose, hemicellulose, and lignin. Instead, peaks representing aromatic C=C stretching (1414 cm^−1^) appeared according to ref. 24.

#### Raman

The distribution and condition of sp^2^-bonded carbon in the microstructure of carbon materials can be assessed using Raman spectroscopy. An understanding of the carbon structure was gained through the Raman spectroscopy analysis of RSB. The RSB displayed two broad bands at 1365 cm^−1^ (D band) and 1595 cm^−1^ (G band), respectively. While the G band represents the oscillation of the C=C bond with the sp^2^ graphitic carbon framework, the D band represents the flaw and imperfection in the carbon substance. The intensity ratio of the *I*_D_/*I*_G_ ratio was 1, which shows the graphitic nature of RSB, as shown in Fig. 4(c).

#### BET and SEM

The BET surface area of RSB was impacted by temperature. The surface area and pore volume of RSB at 700 °C were observed to be 6.37 m^2^ g^−1^ and 7.39 cm^3^ g^−1^ due to cellulose, lignin degradation, and rapid release of H_2_ and CH_4_, resulting in the amorphous carbon structure and micropores.^25^ The average pore diameter of RSB was observed to be 4.80 nm. The SEM image of RSB demonstrates the porous structure, as shown in Fig. 4(e), which is also confirmed by BET results.

## Results and discussion of the supercapacitor study

**Fig. 5:**
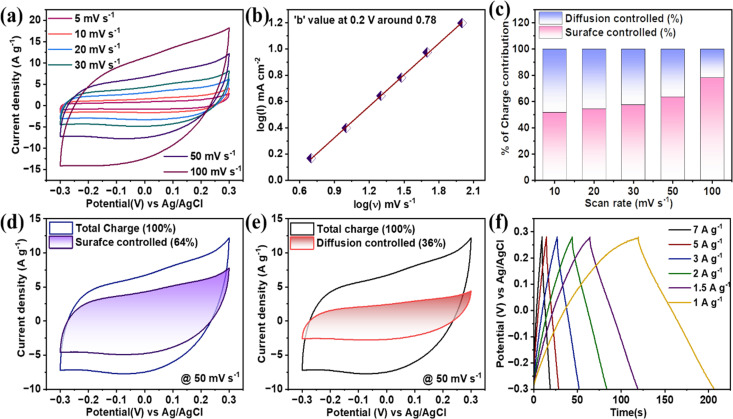
(a) CV curves at various scan rates; (b) ‘*b*’ is calculated at 0.3 V. (c) Percentage of surface controlled and diffusion controlled for different scan rates. (d and e) Surface and diffusion controlled portion over a total current response at 50 mV s^−1^. (f) Charge–discharge at different current rates.

The three-electrode supercapacitive analysis of FCMCM oxide and biochar electrode was carried out in the optimum concentration of potassium hydroxide. As mentioned in the introduction section, the previous studies back the selection of KOH for significant capacitive performance. Small hydrated ionic sizes of K^+^ and OH^−^ ions also facilitate greater ionic conductivity with an easy diffusion process.^26^ Hence, all electrochemical processes are measured using 3 M KOH electrolytes. The curves are represented in Fig. 5(a), which are taken in stable voltage window ranges from −0.3 to 0.3 V. It is obseved from CV curves that neither pure redox kinetics observed prove this high entropy oxide nor a battery kind of material, hence specific capacitance can be expressed as F g^−1^ instead of mA h g^−1^.

On the other hand, the capacity from CV calculated using nonlinear equations does not obey pure double-layer kind of material.^27,28^ Again, the capacitance for this class of materials mainly arises from the surface reactions involved with active electrode materials. These surfaces are mostly surface redox reactions (including surface intercalation/deintercalation), termed pseudocapacitive charge storage characteristics.^29^ Further in higher scanning rate regions, the curves are becoming less pseudocapacitive, directing a certain percentage of electrochemical double-layer characteristics observed with the adsorption of ions on the surface of the active materials. These two characteristics can be further verified with the help of power law that can be expressed as;

1 *i* = *mν*^*n*^

where *i*, *m*, and *n* are current, modifiable variables, respectively, the *n* value ranges between 0.5 to 1.0, with ‘*n*’ closer to 0.5 referring to more faradaic characteristics, while *b* = 1 is called pure capacitive. For the pseudocapacitive high entropy oxide, the slope of the plot log(*i*) *versus* log(*ν*) will give an ‘*n*’ value. The value of *n* here is 0.78, which is an intermediate stage between the pure faradaic and pure capacitive phenomena, proving it to be an excellent supercapacitive material, as shown in Fig. 5(b). Now, these multivalent cations associated with HEO will be a hub for these surface redox activities with the transition between the oxidization states Fe^3+^/Fe^2+^, Co^2+^, Ni^2+^, Mn^4+^/Mn^3+^ during cyclic voltammogram irrespective of scan rates. Now, the above charge storage process can be simplified by two charge storage mechanisms named capacitive effect (*k*_1_*ν*) and slow diffusion controlled effect (*k*_2_*ν*^1/2^).^30^

2 *i*(*ν*) = *k*_1_*ν* + *k*_2_*ν*^1/2^

3
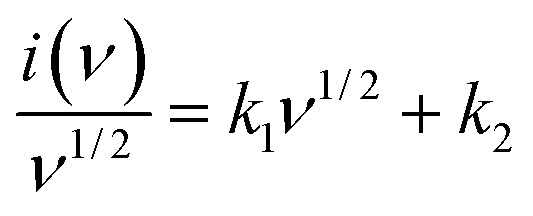
In the above relation, the evaluation of *k*_1_ and *k*_2_ will simplify the relation between the controlled effect and the controlled effect's current response from the total current response. Now, the total voltammetric charge under a voltammogram can be evaluated from the integral area, which is directly related to the scanning rate (*ν*), *Q*(*ν*) = *Q*(total) = *Q*_capacitive_ + *k*_3_*ν*^−1/2^, by plotting the graph between the total charge with respective *ν*^−1/2^, and the *Y*-axis intercept, which is nothing but *Q*_capacitive_, which can be evaluated easily.^31^ Fig. S1† shows the exact value of the calculated surface-controlled effect, which, on further subtraction from the total charge, will give a diffusion-controlled effect. Hence, the relative percentage contribution of *Q*_s_ and *Q*_d_ are shown in Fig. 5(c). It is demonstrated that in lower scanning voltammograms, there is enough time duration available for electrolytic ions to participate in the pseudocapacitive surface redox activities like surface intercalation/deintercalation while increasing the scanning rate, and there will be less pseudocapacitive redox activities. Further, the higher current response in this quinary high entropy oxide electrode is believed to have originated from each transitional oxide; for example, Fe and Mn oxides have more redox activities in the negative potential window, while capacitive materials Ni and Co oxides have redox activities in the positive potential window, with the latter contributing to higher specific capacitance. Fig. 5(d) and (e) show the separation of surface-controlled and diffusion-controlled from the total charge at 30 mV s^−1^.

**Fig. 6:**
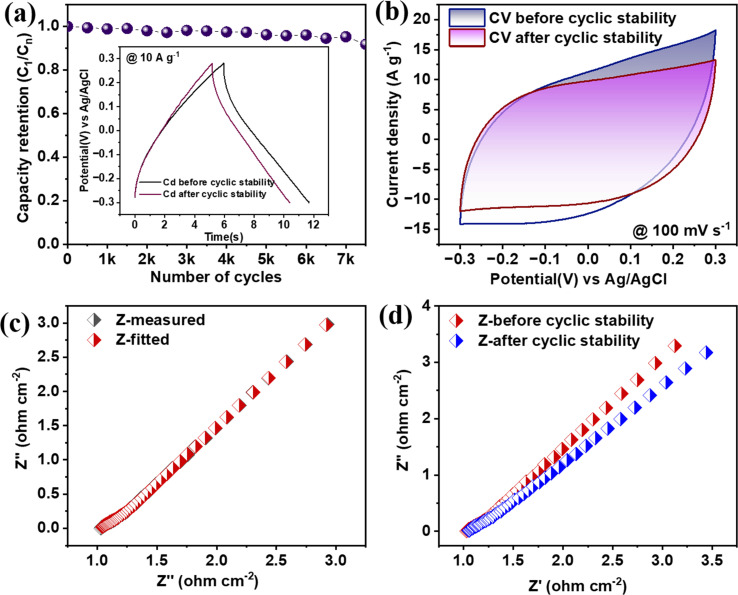
(a) Cyclic stability up to 7500 cycles at 10 A g^−1^, (b) CV before and after stability tests, (c) Nyquist plot, (d) and Nyquist plot before and after stability tests.

Now, the GCD pattern follows the same nonlinear charge–discharge in Fig. 5(f). This HEO electrode shows moderate coulombic efficiency, proving the participation of all quinary transition metallic cations in the electrochemical activities. The pseudocapacitive characteristics will dominate in the lower current rate charging discharging while increasing the current rate surface-controlled characteristics. The following are more ion adsorption characteristics on the active surface of the electrode. The gcd cures follows pseudocapacitive characteristics, the gravimetric capacitance for this curves will be evaluated using an equation as shown in supporting equation data. The highest value of specific capacitance obtained was 313 F g^−1^ for a current rate of 5 mV s^−1^; while increasing the current density to 200, the capacitance was obtained at around 96 F g^−1^. In addition, electrochemical impedance spectroscopy is also measured in between the frequency range of 1 Hz to 10 kHz. The cyclic stability of this HEO electrode three-electrode measurement is verified with the help of a current density of 10 A g^−1^ up to 7500 incessant charge–discharge cycles. As represented in Fig. 6(a), in the initial 2000 cycles, capacity retention will continuously decrease. However, on further increasing the cycles, it remains stable at 92% even after 7500 cycles, which signifies an excellent supercapacitive material for long-term practical durability. This FeCoNiCuMn HEO can be compared with existing literature reports on the high entropy-based supercapacitors such as (FeCoCrMnNi)_3_O_4,_^32^ (FeCoCrMnZn)_3_O_4_,^33^ and (CrMnFeCoNi)_3_O_4_,^34^ which demonstrated the specific capacitance of 332.2 F g^−1^ at 0.3 A g^−1^, 340.3 F g^−1^ at 0.5A g^−1^, 239 F g^−1^ at 0.5A g^−1^, respectively. Recently, Zhang *et al.*^35^ synthesized (FeCoCrMnNi)_3_O_4_ by dealloying techniques, showing 639 F g^−1^ at 1 A g^−1^ in 3 M NaOH but with poor cyclic stability just up to 1000 cycles. In addition, various high entropy composites such as rHEO-CNT,^36^ HEO/f-CSAC,^37^ NiCuFeCoMn-carbonate, HEA-NP@MOL/HCPC display specific capacitances of 157.5 F g^−1^ at 1 A g^−1^, 147.5 F g^−1^ at 1 A g^−1^, 1241 F g^−1^ at 3 A g^−1^, 203 F g^−1^@1 mA cm^−2^ respectively. Comparison of various HEA-based materials with the prepared high entropy oxide electrode Table 1. The above literature analysis shows that this economical induction of melted and ball-milled high entropy oxide emerges as an excellent supercapacitive material irrespective of composite combination or synthesis protocol for high entropy alloy-based materials. Fig. 6(c) shows the Nyquist plot with fitted data with *R*_s_ and CPE with no change of Nyquist plot shape behavior after stability while Fig. 6(d) shows nyquist plot before and after the stability.

**Table 1:** Comparison of various HEA-based materials with the prepared high entropy oxide electrode

Electrode material	Synthesis method	Morphology	Electrolyte	Potential window	Specific capacitance	Ref.
(CrMnFeCoNi)_3_O_4_	Co-precipitation	Quasi spherical	2 M KOH	0.0 to 0.45 V	239 F g^−1^ @0.5 A g^−1^	34
(FeCoCrMnZn)_3_O_4_	Solid state reaction	Irregular particles and agglomeration	1 M KOH	0.15 to 0.5V	340.3 F g^−1^ @0.5 A g^−1^	33
AlCoCrFeNi two phase dissolution	Selective phase dissolution	Nanoporous structure	2 M KOH	−0.2 to 0.5V	700 mF cm^−2^ at 1 mA cm^−2^	8
FeNiCoMnMg HEA-NPs/ACNFs	CTS method	Nanoporous structure	6 M KOH	0.0 to 0.8V	203 F g^−1^@1 mA cm^−2^	7
HEA-NP@MOL/HCPC	Liquid phase synthesis	Cluster of nanoparticles	1 M KOH	−1.0 to 0.0V	495.4 F g^−1^ at 0.5 A g^−1^	16
rHEO-CNT	Solgel method	Connected nanoparticles in nanotubes	1 M H_2_SO_4_	0.0 to 1.0 V	157.5 F g^−1^ at 1A g^−1^	36
HEA-nitrides	Mechanochemical assisted synthesis	Nano flakes architecture	1 M KOH	−1.0 to 0.0 V	230 F g^−1^ at 10 mV s^−1^	38
(TiNbTaZrHf)C powder	Facile electrochemical process	Spherical nanoparticles	1 M KOH	−1.0 to 0.0 V	95.2 F g^−1^ at 10 mV s^−1^	39
(VNbTaZrHf)C	Direct electro-deoxidation	Dense block structure	1 M KOH	−0.7 to −0.2 V	151 F g^−1^ at 10 mV s^−1^	40
HEO/f-CSAC	Grinding	Cavity-type microstructure	1 M NaCl	0.0 to 1.0 V	147.5 F g^−1^ at 1 A g^−1^	37
(Zr_0.5_Ti_0.5_Ce_0.5_Hf_0.5_)O_7_	Sol–gel synthesis	Roughly rice-like	1 M Na_2_SO_4_	−1.0 to 1 V	703.3 F g^−1^ at 1 A g^−1^	41
NiCuFeCoMn-carbonate	Hydrothermal method	Structure flower-like	1 M KOH	0.1 to 0.5 V	1241 F g^−1^ at 3 A g^−1^	42
La_0.7_Bi_0.3_Mn_0.4_Fe_0.3_Cu_0.3_O_3_ HEP	Solvothermal	Spherical shell pore structure	6 M KOH	−1.0 to 0.0 V	480.95C g^−1^ at 0.5 A g^−1^	43
K(MgMnFeCuNi)Fe(CN)_6_	Mechanochemical	Agglomeration	1 M Na_2_SO_4_	0.2 to 1.0 V	175 F g^−1^ at 5 mVs^−1^	44
(FeCoNiCuMn)_3_O_4_	Induction melting, ball milling, annealing	Agglomeration of nanoparticle	3 M KOH	−0.3 to 0.3 V	313 F g^−1^ at 5 mV s^−1^	This work

**Fig. 7:**
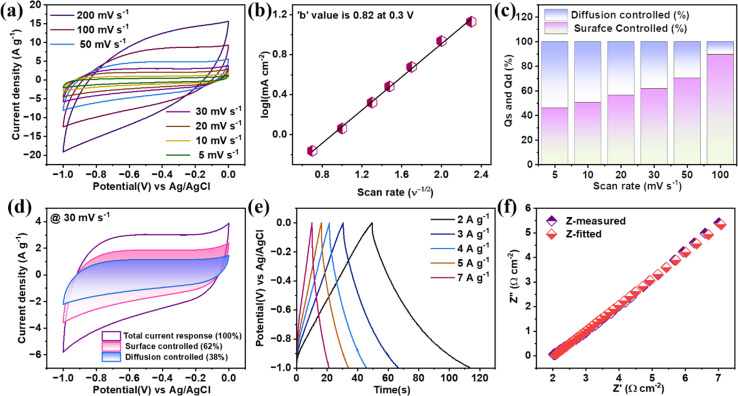
(a) CV at different scanning rates, (b) ‘*b*’ value at −0.3 V, and (c) distinction of surface and diffusion controlled kinetics to the total charge. (d) Amount of charge in the respective CV, (e) GCD at different current densities, and (f) Nyquist plot.

The same electrochemical protocol was followed for the green carbon derived from agricultural waste; three electrode measurements demonstrated the same electrolyte concentration of KOH with an active material mass loading of 1 mg. The cyclic voltammetry curves are measured with a stable potential window of −1.0 to 0.0 V against Ag/AgCl between scan rate ranges from 5 to 200 mV s^−1^. As shown in Fig. 7(a), CV curves, the plot log(*i*) *versus* log(*ν*) will give an ‘*n*’ value. The value of *n* here is 0.82, which is the intermediate stage between the pure faradaic and pure capacitive phenomena but closer to the capacitive behavior of green carbon electrode, which is purely EDLC type Fig. 7(b).

Moreover, gcd plots are also recorded between the 1 A g^−1^ to 7 A g^−1^, as shown in Fig. 7(e). The CV curves are mostly inclined towards the rectangular, while in the lower scanning region, a little edge of pseudocapacitive characteristics is observed. Now, quantifying surface-controlled characteristics from the total charge plot, as shown in supporting Fig. S2(a)†, can quantify both mechanisms. Fig. 7(c) connects the percentage of surface and diffusion kinetics charges with total charges. This will give more ability to understand how surface contribution dominated over increased scanning rate. For a scanning rate of 30 mV s^−1^, the percentage contribution of *Q*_s_ and *Q*_d_ is 62% and 38%, respectively, as shown in Fig. 7(d). The evaluation of specific capacitance at 1 A g^−1^ shows 126 F g^−1^, while at a current rate of 7 A g^−1^, the capacitance decreases to 61.73%. The higher CV areas in the green carbon electrode's negative potential window (−1.0 to 0.0) suggest an excellent negative electrode material for supercapacitor application. Fig. 7(f) shows the Nyquist plot for green carbon electrodes. Fig. S2(b) and (c)† shows the cyclic stability of the biochar electrode at 7 A g^−1^ up to 7500 cycles, green carbon electrode, an initial and final GCD also attached. Fig. S2(d)† shows the initial and final Nyquist plots. A comparison of RS-biochar with various biochar-based supercapacitors from agricultural waste is shown in Table S1.†

### Study of asymmetric liquid state devices

The supercapacitor characteristics of both FeCoNiCuMn HEO and biochar-C can be further analyzed when there is an asymmetric supercapacitive combination. The asymmetric devices have significant importance due to their high specific energy, wide voltage window, and high cyclic stability. Hence, the asymmetric device is demonstrated with FCNCM oxide as the positive and biochar-C as the negative electrodes, respectively. The maximum device efficiency will be observed when charge balance loading is employed on respective electrodes.^45,46^*q*^+^ = *q*^−^*m*^+^*V*^+^*Cp*^+^ = *m*^−^*V*^−^*Cp*^−^

The required mass loading is evaluated at 50 mV s^−1^ with a voltage window of −0.3 to 0.3 (HEA oxide) and −1.0 to 0.0 V (biochar) with a specific capacitance of 250 F g^−1^ and 151.36 F g^−1^; putting these values in the above charge balance equation, the ratio of 0.86 is obtained. This implies that 1 mg of biochar and 0.86 mg of HEA oxide coated on a graphite sheet enables the desired asymmetric electrodes. This asymmetric device was tested in an aqueous 2 M potassium hydroxide solution. Various mass ratios are considered for further optimization; the HEO : biochar ratios of 0.5 : 1, 1 : 1, 1 : 0.5 and 0.85 : 1 are considered in mg, and different cells are constructed, as shown in Fig. S4–S7.†

**Fig. 8:**
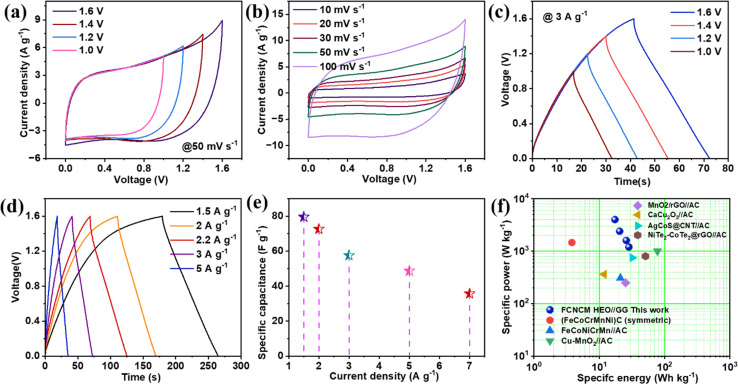
(a) Stability of CV at 50 mV s^−1^ for the asymmetric device. (b) CV at different scan rates. (c) Stability of charge–discharge at 3 A g^−1^. (d) Charge–discharge at different current rates. (e) Specific capacitance *vs.* current density. (f) Ragone plot for the asymmetric device.

Starting with Fig. 8(a), the voltage range stability of this device tested at 50 mV s^−1^ for various voltage points ranges from 1.0 V to 1.6 V, and it is observed that there is no disintegration of CV over increasing voltage, proving a wide voltage stability through asymmetric combination. Next, the CV curves are taken in different scanning rates ranging from 10 to 200 mV s^−1^, as shown in Fig. 8(b) with a voltage window of 0.0 to 1.6 V. The curves seem quasi-rectangular, miming the pseudocapacitive characteristics through the asymmetric combination. Again, more linearity was observed in the lower scan rate, which easily matched the three electrode characteristics. The dominance of slow diffusion-controlled faradaic characteristics results in nonrectangular CVs with the increasing scanning rate; the dominance of surface-controlled characteristics is observed with less surface redox phenomena. These supercapacitive asymmetric characteristics are also observed in the charge–discharge phenomena. As shown in Fig. 8(c), the voltage stability was also tested for gcd curves at a current rate of 3 A g^−1^ with a voltage window of 0.0 to 1.6 V. The curves are continuous with nonrectangular characteristics, proving significant asymmetric aqueous supercapacitive device stability. Hence, all gcd traces are recorded with the help of a current rate ranging from 0.5 to 10 A g^−1^, as shown in Fig. 8(d). Charge discharge traces associated with the lower current rate become more plateau-like with slow diffusion-controlled surface redox characteristics of surface intercalation activities.

In contrast, in the higher current rate region, the curves become less faradaic with more triangular characteristics. From the nonlinear characteristics of charge–discharge, the specific capacitance value is calculated at 2 A g^−1^ as 83 F g^−1^, further lofty on the current rate, it drops to 56% of the initial value, as shown in Fig. 8(e). With this capacity excellence, evaluating the respective specific energy and power from the discharge portion for the respective scan rate is very convenient. Now, this hybrid asymmetric device's highest specific energy of 33.4 W h kg^−1^ is obtained by consuming a specific power of 1700 W kg^−1^. The obtained specific energy can be more comparable with various liquid state asymmetric devices, as shown in Fig. 8(f). Among the different liquid state devices, (FeCoCrMnNi)C^47^ (high entropy carbide symmetric) shows 3.8 W h kg^−1^ at a specific power of 1466.7 W kg^−1^ for the 1 M KOH electrolyte, FeCoNiCrMn//AC^30^ (asymmetric) shows 21 W h kg^−1^ at 307 W kg^−1^ for 3 M KOH electrolyte, microwave synthesized Cu-doped MnO_2_ with asymmetric combination of activated carbon^48^ shows 77.78 W h kg^−1^ at a power density of 1000 W kg^−1^ for 1 M Na_2_SO_4_ electrolyte, MnO_2_/rGO//AC shows 25.14 W h kg^−1^ at 250 W kg^−1^, CaCu_2_O_3_//AC^49^ shows 11.8 W h kg^−1^ at 362.5 W kg^−1^, AgCoS@CNT//AC^50^ shows 32 W h kg^−1^ at 750 W kg^−1^, and the NiTe_2_–Co_2_Te_2_@rGO//AC^51^ HSC device shows a specific capacitance of 51 W h kg^−1^ at 800 W kg^−1^ for 1 M KOH electrolyte. Additionally, specific energy and specific power of different mass loading electrodes are analyzed in detail Fig. S8.† For mass rations, we have analyzed the log(specific energy) *vs.* log(specific power) plot according to the equation, log(specific energy) = *b* log(specific power) + *a*. All four types of mass ratio loadings are 1 : 0.5, 1 : 1, 0.5 : 1, 0.85 : 1, respectively, of HEO and biochar. For HEO : biochar ratio, 1 : 1, slope −0.51, intercept = 2.91 and *R*^2^ value obtained is 0.94; for ratio 0.5 : 1, slope = −0.23, intercept = 2.31 and *R*^2^ value obtained is 0.92; for the ratio 1 : 0.5, slope = −0.17, intercept = 1.94 and *R*^2^ value obtained is 0.93; and for the ratio 0.85 : 1, slope = −0.149, intercept = 1.49 and *R*^2^ value obtained is 0.94. Hence, compared to other mass ratios, clearly, the rate of decrease of energy density with an increase of power density is lesser in the mass ratio of 0.85 : 1.

After evaluating the specific energies for this aqueous asymmetric device, cyclic stability is tested at 5 A g^−1^ up to 7500 cycles Fig. S3(a).† The initial decay obtained for the device was until stable interaction was formed with HEA and KOH electrolyte. With the remaining cycles, capacity retention remains stable at up to 3000 and slowly decays for 7500 cycles.

**Fig. 9:**
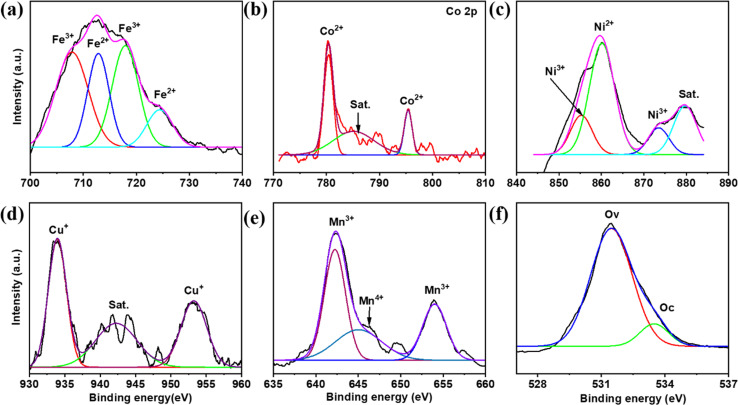
XPS analysis after cyclic stability test of the (FeCoNiCuMn)_3_O_4_ HEO electrode (a) Fe 2p, (b) Co 2p, (c) Ni 2p, (d) Cu 2p, (e) Mn 2p and O 1s respectively.

After testing the stability of the electrode employed for the X-ray photoelectron spectroscopy analysis, peak positions changed slightly. At the same time, the original oxidation states remain dominant in Fig. 9. Concerning the Fe 2p spectrum, as shown above, the peaks at 707.86, 712.84, 717.92, and 724.41 eV correspond to Fe^3+^, Fe^2+^, Fe^3+^, and Fe^2+^, respectively. Similarly, for Co 2p, peaks remain unaltered while the intensity is slightly varied. Next, for Ni 2p, splitting multiple peaks results in Ni^3+^, Ni^2+^, and satellite peaks, respectively. In addition, the Cu 2p peaks are also labeled as Cu^+^, while for Mn 2p, Mn^3+^ and Mn^4+^ are dominant.

Similarly, the rise in oxygen intensity is due to deep interaction with the KOH electrolyte. Further, these multiple peak splitting and intensity changes result in good intercalation and deintercalation of K^+^ and OH^−^ ions with multivalent quinary cations. Moreover, this HEA architecture can also prove the change of states from M^*n*+^ to M^*m*+^. This higher specific capacitance with higher CV response can be plotted as the synergism effect of various elements of the HEA framework.

Enriching the detailed analysis for the FeCoNiCuMn HEO electrode, impedance analysis was employed with cyclic voltammetry and galvanostatic charge–discharge. This impedance spectroscopy is recorded between 10 000 Hz to 1 Hz frequency range with the same electrolytic concentration. Fig. S3(b)–(e)† shows that the Nyquist plot behaves like a pseudocapacitive characteristic with no pure semicircle, proving an excellent pseudocapacitive asymmetric combination. The fitted circuit behaved like a modified Randel's circuit. In addition, the Bode plot and capacitive impedance plot over the frequency range were also calculated.

## Conclusions

The above studied (FeCoNiCuMn)_3_O_4_ spinel type high entropy oxide for supercapacitor applications possesses good potential, and the multivalent oxidation states evolved due to higher specific capacitance. Again, fine mesoporous distributed morphology and agglomerated nanostructure further add a higher impact on supercapacitor properties. Briefly analyze the result of supercapacitor study at 3 M KOH solution around shows a specific capacitance of 313.16 F g^−1^ while for 1 A g^−1^ around 143.33 F g^−1^. In addition, green carbon is also prepared from the catalytic annealing of rice straw biochar, which has good performance in the negative potential window of around 232 F g^−1^ at 5 mV s^−1^ while for 1 A g^−1^, the value is around 126 F g^−1^. Now, using HEO as a positive electrode and green carbon as a negative electrode, the fabricated aqueous asymmetric device shows the highest specificity of 83 F g^−1^ at 2 A g^−1^. Further, their specific energy and power were deconvoluted around 33.4 W h kg^−1^ and 1700 W kg^−1^, respectively. Moreover, synthesizing both HEO and green carbon electrodes from agricultural waste using a green synthesis protocol provides researchers with a wide range of opportunities.

## Data availability

Data will be available on request.

## Conflicts of interest

There are no conflicts of interest to declare.

## Supplementary Material

RA-014-D4RA05204H-s001

## References

[cit1] Zhao H., Liu L., Vellacheri R., Lei Y. (2017). Recent Advances in Designing and Fabricating Self-Supported Nanoelectrodes for Supercapacitors. Adv. Sci..

[cit2] Wang R., Yao M., Niu Z. (2020). Smart supercapacitors from materials to devices. Infomat.

[cit3] Bigdeloo M., Kowsari E., Ehsani A., Chinnappan A., Ramakrishna S., Aliakbari R. (2021). Review on innovative sustainable nanomaterials to enhance the performance of supercapacitors. J. Energy Storage.

[cit4] Shao Y., El-Kady M. F., Sun J., Li Y., Zhang Q., Zhu M., Wang H., Dunn B., Kaner R. B. (2018). Design and Mechanisms of Asymmetric Supercapacitors. Chem. Rev..

[cit5] Hussain I., Lamiel C., Ahmad M., Chen Y., Shuang S., Javed M. S., Yang Y., Zhang K. (2021). High entropy alloys as electrode material for supercapacitors: A review. J. Energy Storage.

[cit6] Qu J., Buckingham M. A., Lewis D. J. (2023). High-entropy materials for electrochemical energy storage devices. Energy Adv..

[cit7] Xu X., Du Y., Wang C., Guo Y., Zou J., Zhou K., Zeng Z., Liu Y., Li L. (2020). High-entropy alloy nanoparticles on aligned electronspun carbon nanofibers for supercapacitors. J. Alloys Compd..

[cit8] Kong K., Hyun J., Kim Y., Kim W., Kim D. (2019). Nanoporous structure synthesized by selective phase dissolution of AlCoCrFeNi high entropy alloy and its electrochemical properties as supercapacitor electrode. J. Power Sources.

[cit9] Yuan Y., Xu Z., Han P., Dan Z., Qin F., Chang H. (2021). MnO2-decorated metallic framework supercapacitors fabricated from duplex-phase FeCrCoMnNiAl0.75 Cantor high entropy alloy precursors through selective phase dissolution. J. Alloys Compd..

[cit10] Zheng Z., Liang B., Gao J., Ren J., Liu Z., Hou X., Sun J., Mei S. (2023). Dielectric properties of (FeCoCrMnZn)3O4 high-entropy oxide at high pressure. Ceram. Int..

[cit11] Das S., Kumar S., Sarkar S., Pradhan D., Tiwary C. S., Chowdhury S. (2024). High entropy spinel oxide nanoparticles for visible light-assisted photocatalytic degradation of binary mixture of antibiotic pollutants in different water matrixes. J. Mater. Chem. A.

[cit12] Miao L.-Z., Guo Y.-X., Liu Z.-Y., Li Y., Zhu J., Wu L. (2023). High-entropy alloy nanoparticles/biochar as an efficient catalyst for high-performance treatment of organic pollutants. Chem. Eng. J..

[cit13] Qiu H.-J., Fang G., Gao J., Wen Y., Lv J., Li H., Xie G., Liu X., Sun S. (2019). Noble Metal-Free Nanoporous High-Entropy Alloys as Highly Efficient Electrocatalysts for Oxygen Evolution Reaction. ACS Mater. Lett..

[cit14] Huang H., Zhao Y., Bai Y., Li F., Zhang Y., Chen Y. (2020). Conductive Metal–Organic Frameworks with Extra Metallic Sites as an Efficient Electrocatalyst for the Hydrogen Evolution Reaction. Adv. Sci..

[cit15] Phakatkar A. H., Saray M. T., Rasul M. G., Sorokina L. V., Ritter T. G., Shokuhfar T., Shahbazian-Yassar R. (2021). Ultrafast Synthesis of High Entropy Oxide Nanoparticles by Flame Spray Pyrolysis. Langmuir.

[cit16] Shen E., Song X., Chen Q., Zheng M., Bian J., Liu H. (2021). Spontaneously Forming Oxide Layer of High Entropy Alloy Nanoparticles Deposited on Porous Carbons for Supercapacitors. Chemelectrochem.

[cit17] Wang S., Hu J., Jiang L., Li X., Cao J., Wang Q., Wang A., Li X., Qu L., Lu Y. (2019). High–performance 3D CuO/Cu flowers supercapacitor electrodes by femtosecond laser enhanced electrochemical anodization. Electrochim. Acta.

[cit18] Huang K., Peng D., Yao Z., Xia J., Zhang B., Liu H., Chen Z., Wu F., Wu J., Huang Y. (2021). Cathodic plasma driven self-assembly of HEAs dendrites by pure single FCC FeCoNiMnCu nanoparticles as high efficient electrocatalysts for OER. Chem. Eng. J..

[cit19] Zhang M., Jiang Z., Niu M., Sun Y., Zhang X. (2022). Tribological behavior of CoCrFeNiMn high-entropy alloy against 304, Al2O3 and Si3N4 counterparts. Wear.

[cit20] Bourke J., Manley-Harris M., Fushimi C., Dowaki K., Nunoura T., Antal M. J. (2007). Do All Carbonized Charcoals Have the Same Chemical Structure? 2. A Model of the Chemical Structure of Carbonized Charcoal. Ind. Eng. Chem. Res..

[cit21] Guerrero M., Ruiz M. P., Millera Á., Alzueta M. U., Bilbao R. (2008). Characterization of Biomass Chars Formed under Different Devolatilization Conditions: Differences between Rice Husk and Eucalyptus. Energy Fuels.

[cit22] Wang H., Chu Y., Fang C., Huang F., Song Y., Xue X. (2017). Sorption of tetracycline on biochar derived from rice straw under different temperatures. PLoS One.

[cit23] Keiluweit M., Nico P. S., Johnson M. G., Kleber M. (2010). Dynamic Molecular Structure of Plant Biomass-Derived Black Carbon (Biochar). Environ. Sci. Technol..

[cit24] Wu W., Yang M., Feng Q., McGrouther K., Wang H., Lu H., Chen Y. (2012). Chemical characterization of rice straw-derived biochar for soil amendment. Biomass Bioenergy.

[cit25] Zhao B., O'Connor D., Zhang J., Peng T., Shen Z., Tsang D. C. W., Hou D. (2018). Effect of pyrolysis temperature, heating rate, and residence time on rapeseed stem derived biochar. J. Clean. Prod..

[cit26] Yang D. and Ionescu M. I., Metal Oxide–Carbon Hybrid Materials for Application in Supercapacitors, 2017, 10.1016/b978-0-12-810464-4.00008-5

[cit27] Gogotsi Y., Penner R. M. (2018). Energy Storage in Nanomaterials – Capacitive, Pseudocapacitive, or Battery-like?. ACS Nano.

[cit28] Gogotsi Y. (2014). What nano can do for energy storage. ACS Nano.

[cit29] Wang Y., Song Y., Xia Y. (2016). Electrochemical capacitors: mechanism, materials, systems, characterization and applications. Chem. Soc. Rev..

[cit30] Mohanty G. C., Gowda C. C., Gakhad P., Sanjay M., Sarkar S., Biswas K., Singh A., Tiwary C. S. (2023). High energy density liquid state asymmetric supercapacitor devices using Co–Cr–Ni–Fe–Mn high entropy alloy. Mater. Adv..

[cit31] Mohanty G. C., Gowda C. C., Gakhad P., Das S., Sanjay M., Chowdhury S., Biswas K., Singh A., Tiwary C. S. (2023). Iron-Cobalt-Nickel-Copper-Zinc (FeCoNiCuZn) high entropy alloy as positive electrode for high specific capacitance supercapacitor. Electrochim. Acta.

[cit32] Yin Y., Zhang W.-B., Zhang X.-L., Theint M. M., Yang J.-L., Yang Z.-Q., Li J.-J., Liang S., Ma X.-J. (2023). Low Dimensional High Entropy Oxide (FeCoCrMnNi)3O4 for Supercapacitor Application. Dalton Trans..

[cit33] Liang B., Ai Y., Wang Y., Liu C., Ouyang S., Liu M. (2020). Spinel-Type (FeCoCrMnZn)3O4 High-Entropy Oxide: Facile Preparation and Supercapacitor Performance. Materials.

[cit34] Talluri B., Aparna M. L., Sreenivasulu N., Bhattacharya S. S., Thomas T. (2021). High entropy spinel metal oxide (CoCrFeMnNi)3O4 nanoparticles as a high-performance supercapacitor electrode material. J. Energy Storage.

[cit35] Zhang D., Xu S., Li T., Zhang M., Qi J., Wei F., Meng Q., Ren Y., Cao P., Sui Y. (2023). High-Entropy Oxides Prepared by Dealloying Method for Supercapacitors. ACS Appl. Eng. Mater..

[cit36] Lal M. S., Sundara R. (2019). High Entropy Oxides—A Cost-Effective Catalyst for the Growth of High Yield Carbon Nanotubes and Their Energy Applications. ACS Appl. Mater. Interfaces.

[cit37] Lal M. S., Sundara R. (2022). Multifunctional high entropy oxides incorporated functionalized biowaste derived activated carbon for electrochemical energy storage and desalination. Electrochim. Acta.

[cit38] Jin T., Sang X., Unocic R. R., Kinch R. T., Liu X., Hu J., Liu H., Dai S. (2018). Mechanochemical-Assisted Synthesis of High-Entropy Metal Nitride via a Soft Urea Strategy. Adv. Mater..

[cit39] Sure J., Sri Maha Vishnu D., Kim H., Schwandt C. (2020). Facile Electrochemical Synthesis of Nanoscale (TiNbTaZrHf)C High-Entropy Carbide Powder. Angew. Chem., Int. Ed..

[cit40] Yang Y., Chen B., Chen J., Hu L., Hu M. (2022). Preparation of (VNbTaZrHf)C high-entropy carbide nanoparticles via electro-deoxidation in molten salt and their supercapacitive behaviour. Can. Metall. Q..

[cit41] Tang P., Cao Y., li H., Lu M., Qiu W. (2022). The preparation of high-performance aqueous supercapacitor with high-entropy pyrochlore-type electrode and super-concentrated electrolyte. Ceram. Int..

[cit42] Verma A., Kim K. H., Mathur S., Lee D. (2022). Interdependence of the electrical performance of NiCuFeCoMn multi-structure carbonates as electrode material for supercapacitors. J. Alloys Compd..

[cit43] Nan H., Lv S., Xu Z., Feng Y., Zhou Y., Liu X., Liu M., Wang T., Hu X., Tian H. (2022). Inducing the Cocktail Effect in Yolk-Shell High-Entropy Perovskite Oxides Using an Electronic Structural Design for Improved Electrochemical Applications. SSRN Electron. J..

[cit44] Jiang W., Wang T., Chen H., Suo X., Liang J., Zhu W., Li H., Dai S. (2021). Room temperature synthesis of high-entropy Prussian blue analogues. Nano Energy.

[cit45] Mohanty G. C., Chowde Gowda C., Gakhad P., Verma A., Das S., Chowdhary S., Bhattacharya J., K Singh A., Biswas K., Tiwary C. S. (2024). Enhanced energy density of high entropy alloy (Fe-Co-Ni-Cu-Mn) and green graphene hybrid supercapacitor. Energy Storage.

[cit46] Noori A., El-Kady M. F., Rahmanifar M. S., Kaner R. B., Mousavi M. F. (2019). Towards establishing standard performance metrics for batteries, supercapacitors and beyond. Chem. Soc. Rev..

[cit47] Zhang X.-L., Zhang W.-B., Yin Y., Theint M. M., Guo S.-B., Chai S.-S., Zhou X., Ma X.-J. (2023). Sol–gel method preparation and high-rate energy storage of high-entropy ceramic (FeCoCrMnNi)C porous powder. Ceram. Int..

[cit48] Jangu S., Kumar S., Deepika K. N., Jacob C., Pradhan D. (2023). Effect of Microwave Power and Cu Doping on MnO 2 Nanostructures and Its Supercapacitor Performance. ACS Appl. Electron. Mater..

[cit49] Veerapandi G., Prabhu S., Ramesh R., Govindan R., Sekar C. (2022). Pseudo spin-ladder CaCu2O3 nanostructures as potential electrode material for asymmetric supercapacitors. J. Energy Storage.

[cit50] Al Ojeery A., ul Hassan H., Al Balawi S. A., Iqbal M. W., Afzal A. M., Hadia N. M. A. (2023). Growth of AgCoS@CNTs composite on nickel foam to enrich the redox active sites for battery-supercapacitor hybrid energy storage device. J. Phys. Chem. Solids.

[cit51] Farshadnia M., Ensafi A. A., Mousaabadi K. Z., Rezaei B., Demir M. (2023). Facile synthesis of NiTe2-Co2Te2@rGO nanocomposite for high-performance hybrid supercapacitor. Sci. Rep..

