# Supplementary material for: Synthesis of (FeCoNiCuMn)3O4 spinel-high entropy oxide and green carbon from agricultural waste for supercapacitor application
Source: RSC Adv. 2024 Oct 24;14(46):33830–42. doi: 10.1039/d4ra05204h (PMC11500537; doi:10.1039/d4ra05204h)
Supplement: RA-014-D4RA05204H-s001 [file RA-014-D4RA05204H-s001.pdf]

Supplementary Information (SI) for RSC Advances.
This journal is © The Royal Society of Chemistry 2024

Synthesis of $(FeCoNiCuMn)_3O_4$ spinel-High entropy oxide and, green carbon from agricultural waste for supercapacitor application.

Gobinda Chandra Mohanty[1], Amlan Asish[2] Shubhasikha Das[3] Anu Verma[3]

[1]School of Nano science and Technology, Indian Institute of Technology, Kharagpur, West Bengal India 721302.

2Advanced Technology Development Centre (ATDC), Indian Institute of Technology, Kharagpur, Kharagpur-721302, India.

[3]School of Environmental Science and Technology, Indian Institute of Technology, Kharagpur,

West Bengal, India

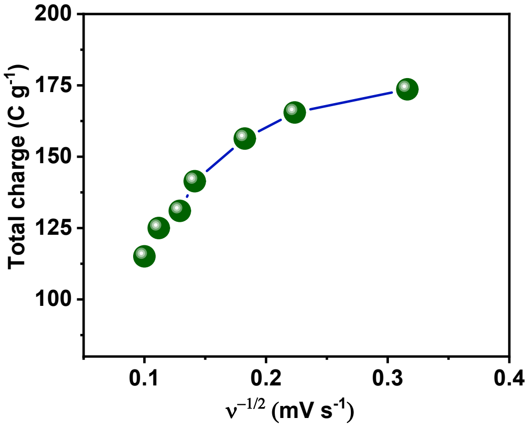

Figure S1: Total charge(Q) vs inverse square root of scan rate $v^{-1/2}$.

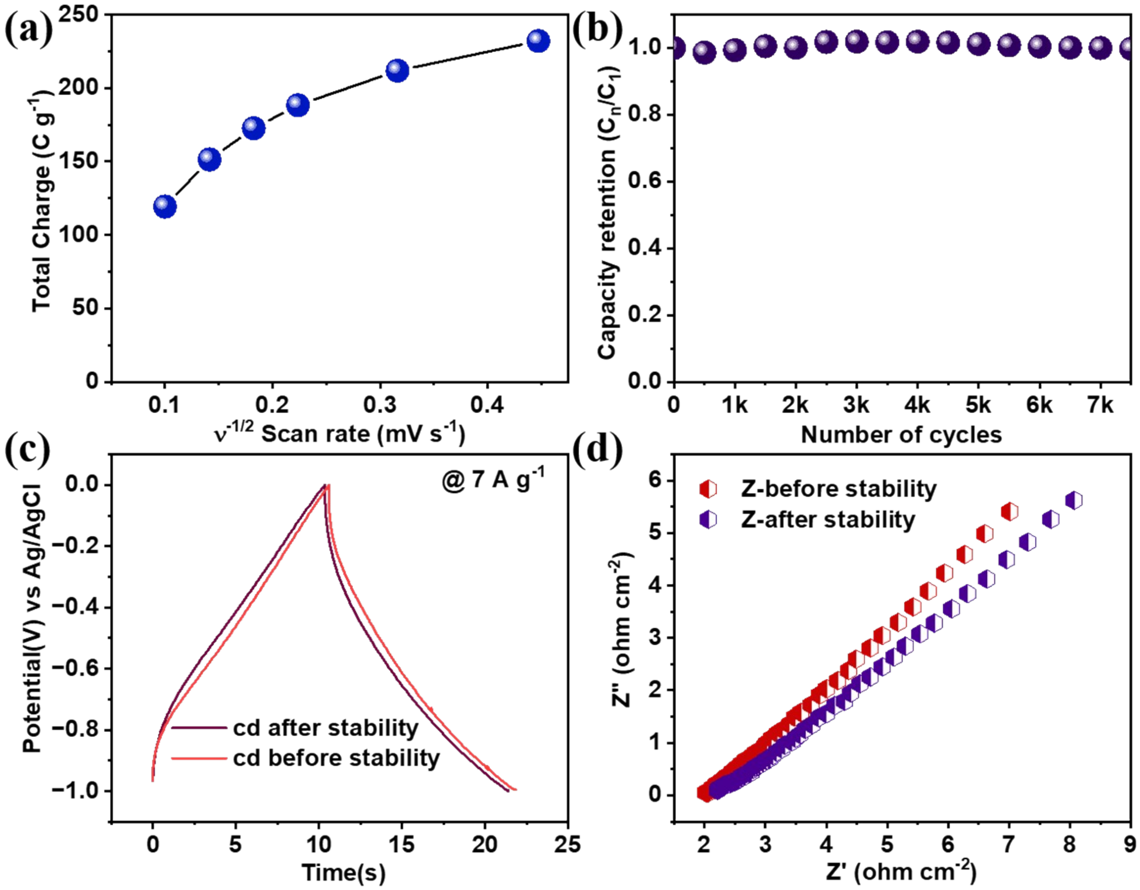

Figure S2: (a) Total charge(Q) vs inverse square root of scan rate $v^{-1/2}$,(b) Cyclic stability at 7 A g^{-1}, (c) GCD at 7 A g^{-1} before and after stability, (d)EIS before and after cyclic stability of biochar electrode.

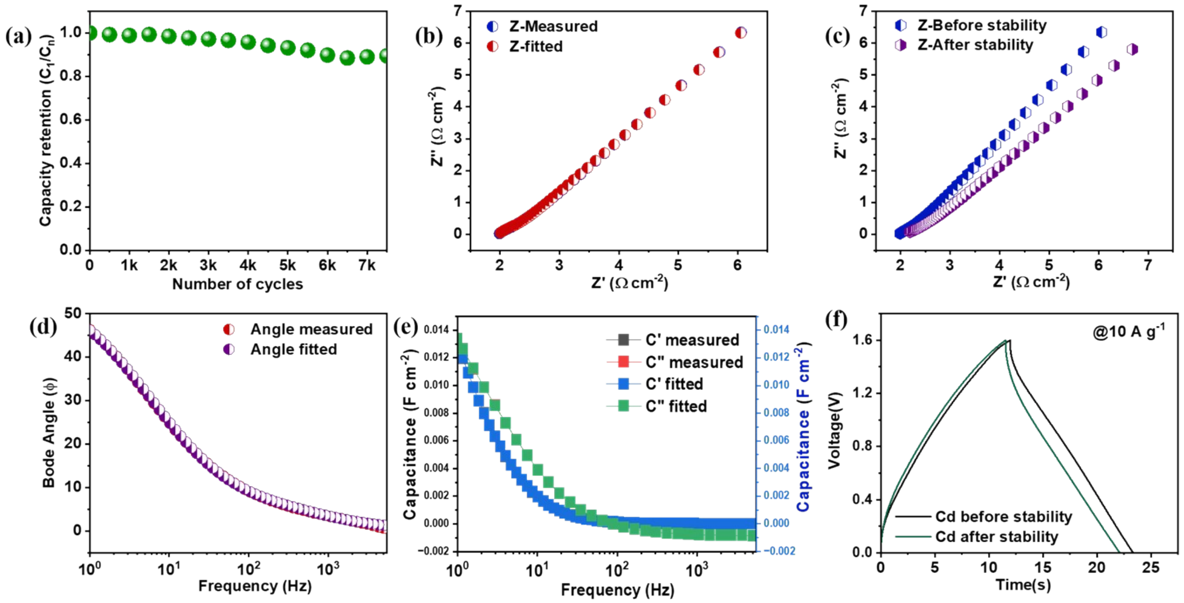

Figure S3: (a) Cyclic stability of ASc device, (b) Nyquist plot of device, (c) EIS of Nyquist plot before and after stability, (d) Bode plot, (e) Capacitance plot and (f) GCD of device at 10 A g^{-1} after and before cyclic stability.

different mass loading ratios are taken for measurement for various asymmetric cells.

Ratio 1: 0.5 mg of HEO and 1 mg of biochar:

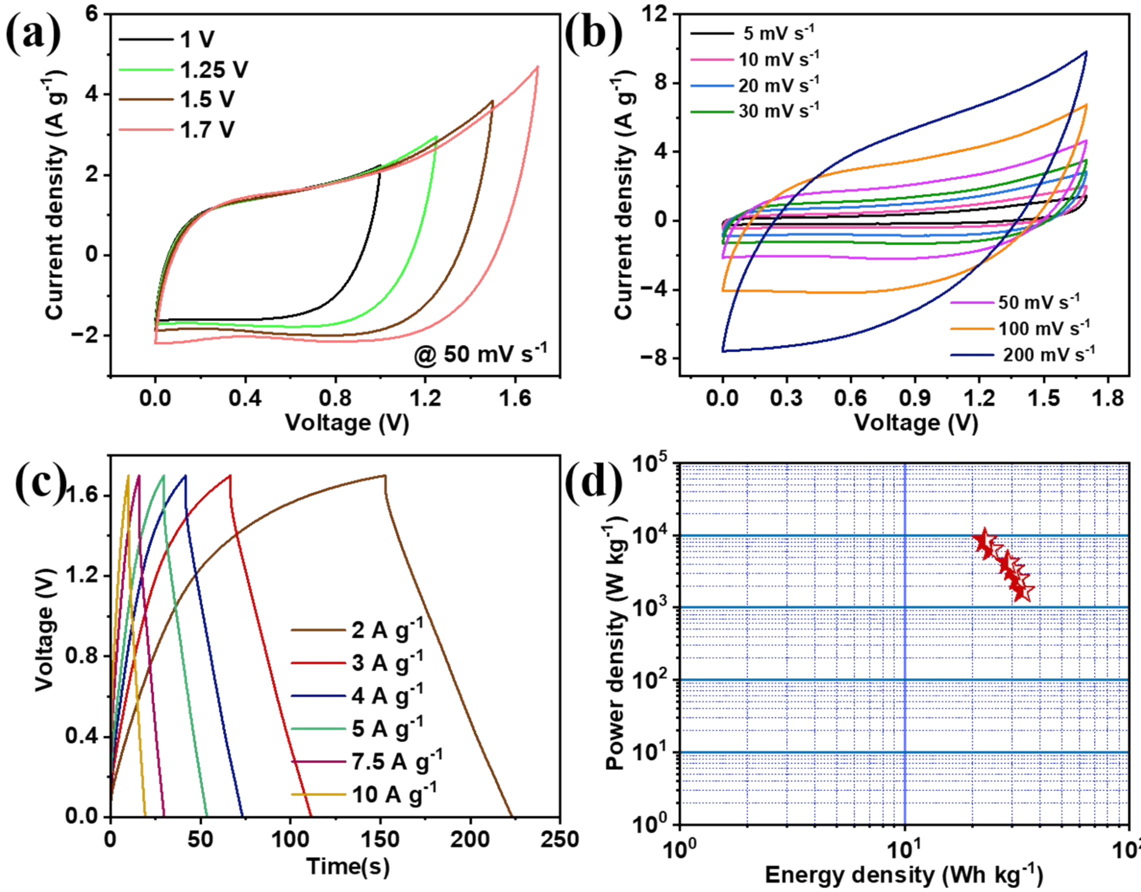

Figure S4: (a) CV stability checked, (b) CV at various scan rate, (c) GCD curves at various current rate, and (d) Ragone plot for asymmetric device.

Ratio 2: 1 mg of HEO and 1 mg of biochar:

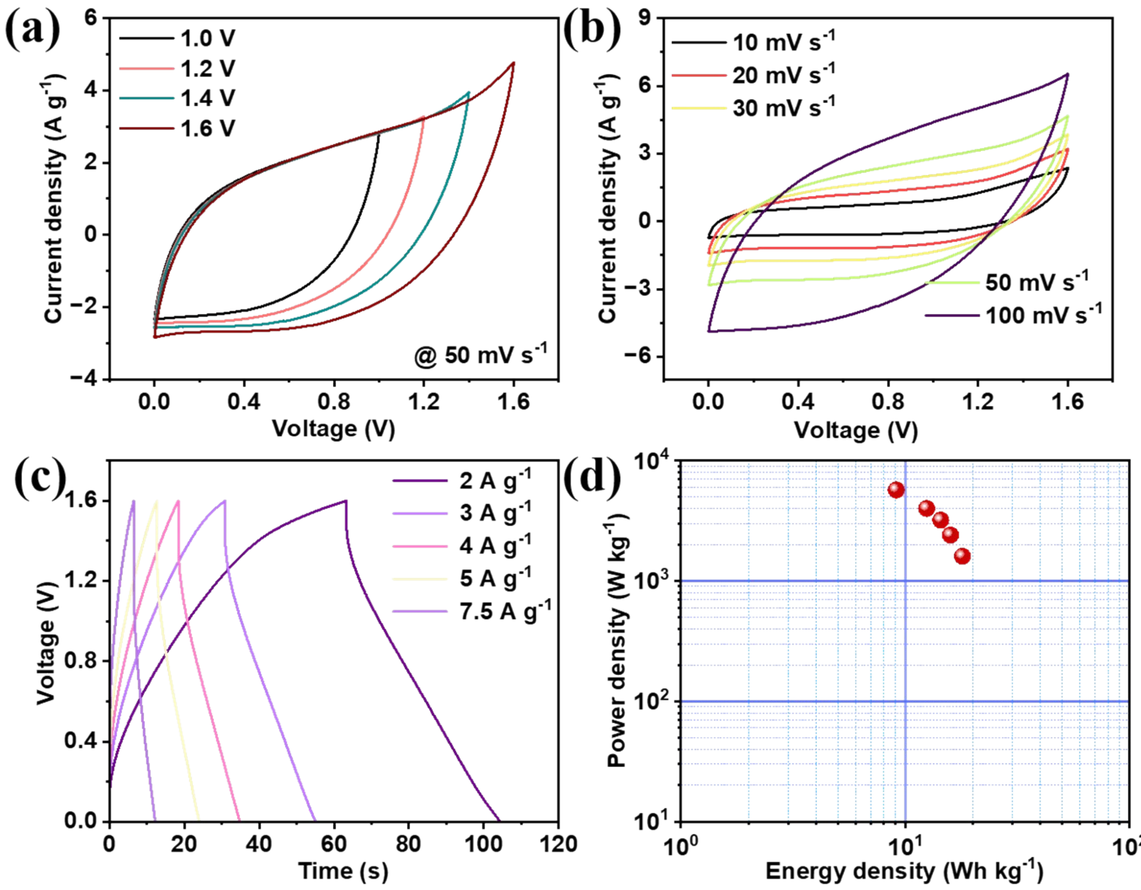

Figure S5: (a) CV stability checked, (b) CV at various scan rate, (c) GCD curves at various current rate, and (d) Ragone plot for asymmetric device.

Ratio 3: 1 mg of HEO and 0.5 mg of biochar:

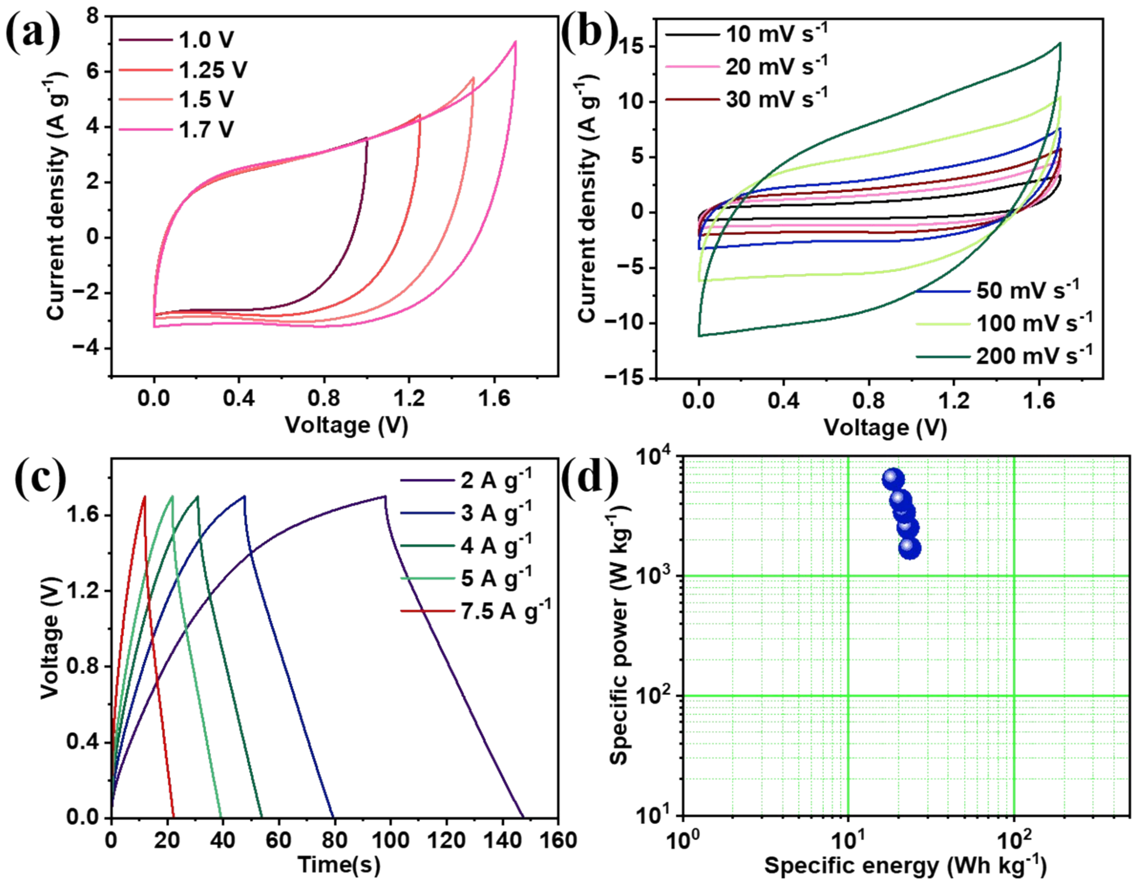

Figure S6: (a) CV stability checked, (b) CV at various scan rate, (c) GCD curves at various current rate, and (d) Ragone plot for asymmetric device.

Ratio 4: 0.85 mg of HEO and 1 mg of biochar:

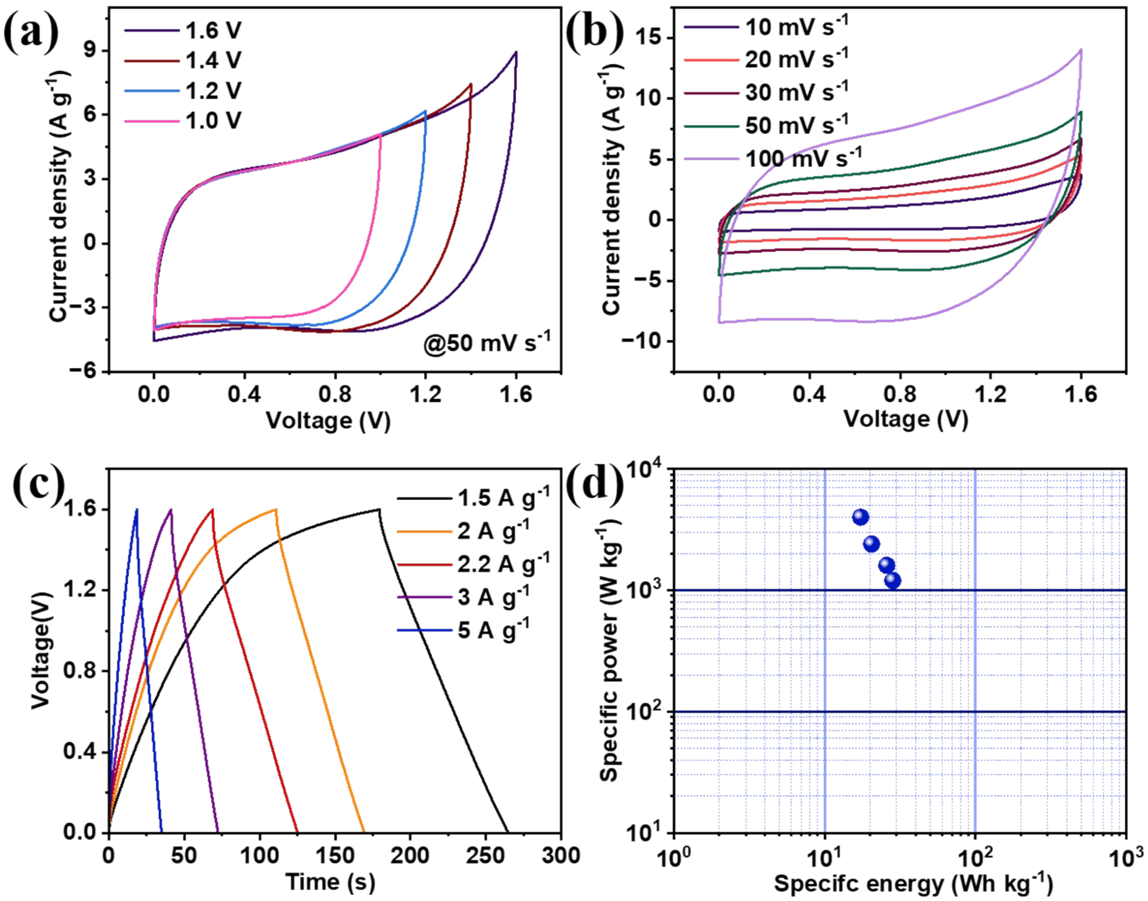

Figure S7: (a) CV stability checked, (b) CV at various scan rate, (c) GCD curves at various current rate, and (d) Ragone plot for asymmetric device.

Mass loading ratio<br>HEO: Biochar in mg	Specific energy(Wh kg^{-1})	Specific Power(W kg^{-1})
0.5 : 1	33.4	1700
1 : 1	17.82	1600
1 : 0.5	23.44	1700

0.85 : 1	33.4	1700

Further in depth analyzing the specific energy as well as specific power from reference[1] we can further analyze as follows:

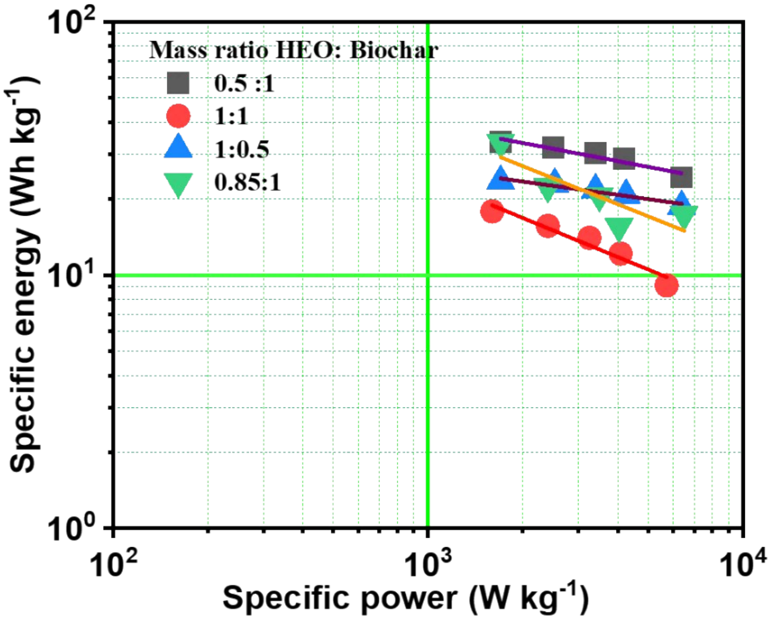

FigureS8: Specific energy vs specific power at different mass loading.

Additionally, specific energy and specific of different misloading are analyzed in detail. For mass rations, we have analyzed the log(specific energy) vs log(specific power) plot according to the equation log(specific energy) = b log(specific power) + a. For all four types of mass ratio loadings are: 1:0.5, 1:1, 0.5:1, 0.85:1 respectively of HEO and biochar taken. For HEO: Biochar ratio, 1:1, slope -0.51, intercept = 2.91 and R^2 value obtained 0.94, for ratio 0.5:1 we got slope -0.23, intercept = 2.31 and R^2 value obtained 0.92, for the ratio 1: 0.5 we got slope -0.17, intercept = 1.94 and R^2

value obtained 0.93, and for the ratio 0.85: 1 we got slope -0.149, intercept = 1.49 and R^2 value obtained 0.94. Hence as compare to other mass ratio, clearly indicate rate of decrease of energy density with increase of power density is lesser in mass ratio of 0.85:1.

The morphological characterizations by atomic force microscopy (AFM) reveals that the as-prepared NPs are quasi-spherical, with an average size of 87 nm × 90 nm (**Figure S9** below).

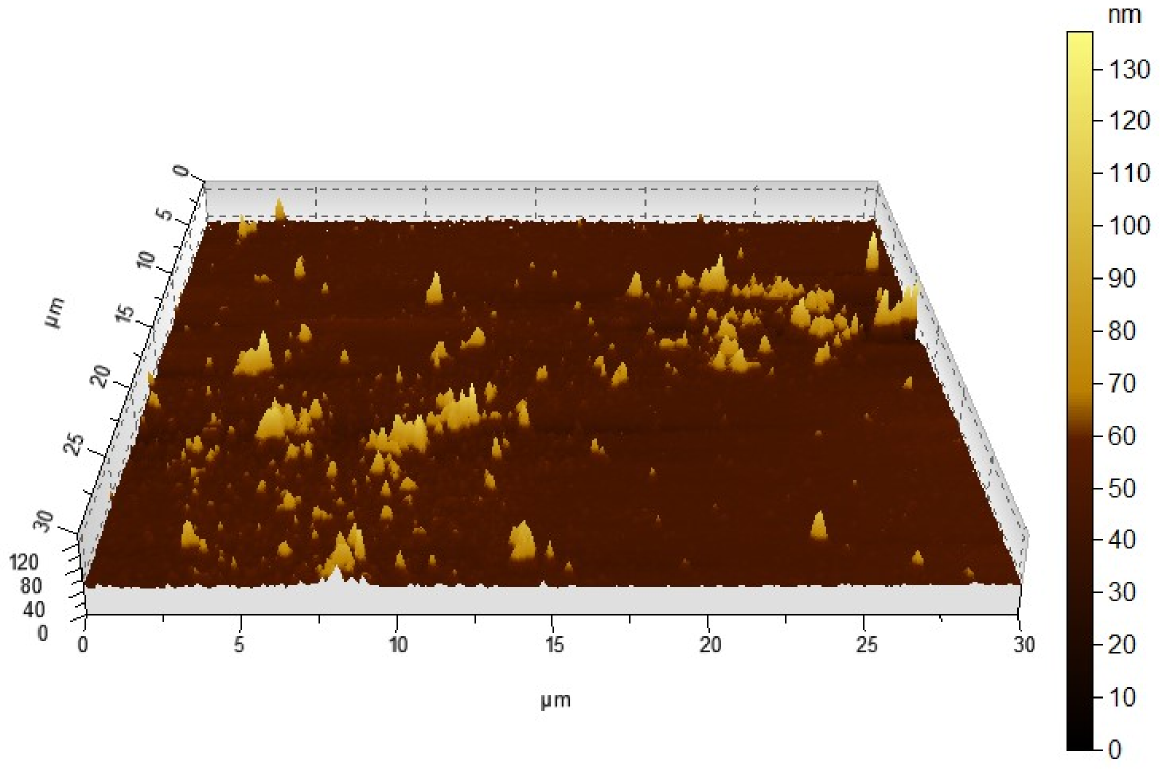

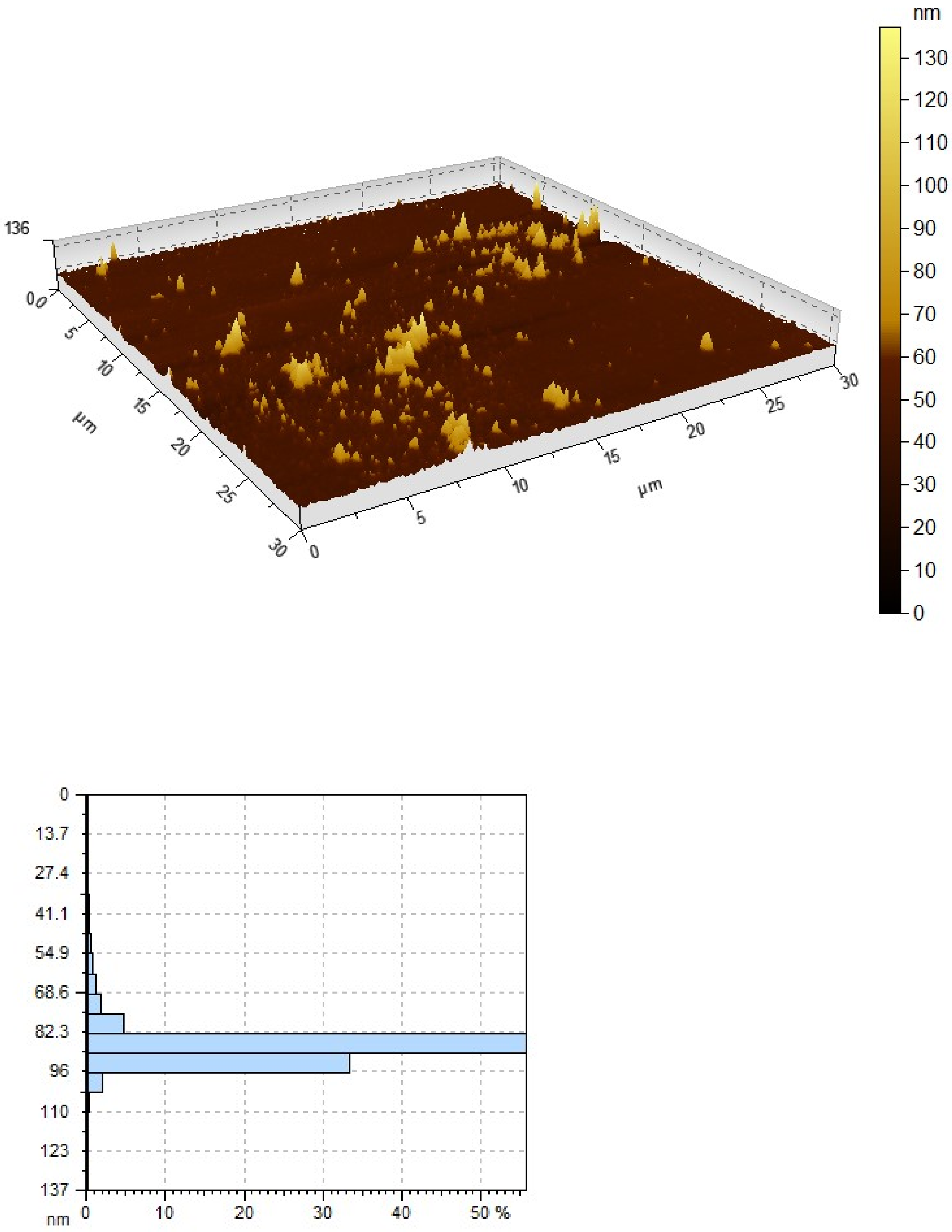

Figure S9: AFM pattern of FCNCM HEO.

Carbon sources	Pyrolysis Activation techniques	Morphology	Electrolyte/ Potential Window (V)	Specific capacitance	Cathode// Anode	Energy Density (Wh/kg)	Power Density (W/kg)	Cyclic stability	Device performance	Device Electrolyte/ Potential Window(V)	Ref.
Peanut shells	Calcined in a furnace 800^0C/KOH	Worm-hole-like pores	1M KOH -0.5 to 0.5	136F/g @ 2A/g	-	-	-	-	-	-	[2]
wolfberry fruits	Catalytic carbonization/ $Sncl_2$	Blanket-like rough structure	6M KOH -1.0 to 0.0	365F/g @ 0.2A/g	Symmetric device	23.2	225	96.4% at 10k	209 F/g @ 0.5 A/g	1M Li_2SO_4 0.0 to 1.8	[3]

Table S1: Comparison of RS-biochar with various biochar based supercapacitors from agricultural wastage.

Chestnut pulp	Carbonization/ KOH	pseudo-honeycomb-like 3D network	6M KOH -1.0 to 0.0	373F/g @ 0.5A/g	-	-	-	99.7% at 10k	-	-	4
Cornstalk	800^{0}C Carbonization	three-dimensional mesh-like pore	6M KOH -1.0 to 0.0	350.4F/g @0.2A/g	Symmetric device	10.1	249.9	99.8% at 10k	308 F/g @ 0.2A/g	6M KOH 0.0 to 1.0	5
Wheat straw	Microwave heating 500^{0}C/KOH	interconnected macropores	6M KOH -1.0 to 0.0	325F/g @ 0.5A/g	Symmetric device	21.5	7.2 kW/kg	90.7% at 10k	268.5F/g @0.5A/g	PVA/LiCl -0.4 to 0.4	6
Discarded tea waste	700^{0}C Carbonization	bulk flakes densely packed structure	3M KOH -0.8 to 0.3	131.95F/g @0.5A/g	-	-	-	-	-	-	7
Cherry stones	700^{0}C Carbonization	Large pore structure	6M KOH -1.0 to 0.0	370.5 F/g @ 0.5A/g	-	-	-	99.1% at 5k	-	-	8
Corncob waste	750^{0}C Carbonization/ KOH	Uniform porous structure	6M KOH -1.0 to 0.0	394.9F/g @ 1A/g	-	8.9	2.502 kW/kg	99% at 10k	-	-	9
Wheat husk	800^{0}C Carbonization	Hollow tunnel's structure	6M KOH -1.0 to 0.0	271.5F/g @ 0.5A/g	-	-	-	82% at 5k		-	10
Rice straw	400^{0}C carbonization/ KOH	Mesoporous structure	6M KOH -1.0 to 0.0	324 @0.5A/g	Symmetric	48.9	750	95% at 10k	-	EMI-TFSI (Ionic liquid)	11
Garlic Seeds	700^{0}C Carbonization/ KOH	Porous honeycomb-like structure	6M KOH -1.0 to 0.0	268F/g @0.5A/g	Symmetric	31.7	500	99.2% at 10k	228.2 F/g at 0.5 A/g	6M KOH 1.0 to 0.0	12
Willow Wood	800^{0}C Carbonization	Microporous structure	6M KOH -1.0 to 0.0	394F/g @ 1A/g	Symmetric	23	10 kW/kg	94% at 5k	201 F/g at 0.5 A/g	1M Na_2SO_4 0.0 to 1.8	13
Waste Bagasse	700^{0}C Carbonization/	Porous Structure	6M KOH -1.0 to 0.0	455F/g@ 0.5A/g	Symmetric	22.3	220.9	93.4% at 10k	206F/g at 0.5A/g	1M Na2SO4	14

	KOH									1.0 to 1.8	
Taro epidermis	800^0C Carbonization/ KOH	Cambered sheet structure	6M KOH -1.0 to 0.0	466 @ 1A/g	Symmetric	17.05	50.05	99.8% at 40k	126.6 at 0.1A/g	6M KOH 2.0 to 0.0	[15]
Pine nut shells	600^0C Carbonization/ KOH	interconnected carbon nanosheets	6 M KOH -1.0 to 0.0	-	Symmetric	11.9	463.6	94.6% at 2A/g At 10k	324F/g at 0.05A/g	6M KOH 1.0 to 0.0	[16]
Crab shells and rice husks	700^0C Carbonization/ HCl	3D hierarchically porous structure	6 M KOH -1.0 to 0.0	474 F/g @0.5A/g	Symmetric	20.5	4500	95.6% at 20k	-	1M Na2SO4 1.0 to 1.8	[17]
Bean dregs	500^0C Carbonization/ KOH	Porous Structure	6 M KOH -1.0 to 0.0	197 F/g @0.3 A/g	Symmetric	18.43 $\mu Wh/cm^2$	120 $\mu W/cm^2$	86% at 2.5k	207 mF/cm^2 at $0.3mA/cm^2$	PVA-KOH 0.0 to 0.8	[18]
Pomelo seed	700^0C Carbonization/ KOH	Interconnected quasi-spherical carbon		845 F/g @ 1A/g				93.8% at 10k @10A/g			[19]
Rice straw	600^0C carbonization	2D graphitic structure	3M KOH -1.0 to 0.0.	121.5 F g^{-1} at 1 A g^{-1}	Asymmetric	33.4	1700	90% at 7.5k @ 5 A g^{-1}	83 F g^{-1} at 2 A g^{-1}	3M aqueous KOH	This work

Reference:

(1) Basak, U.; Das, A.; Maity, S.; Chatterjee, D. P.; Nandi, A. K. Polyaniline-(Dinonylnapthyl Disulphonic Acid) Hydrogel Showing Enhanced Supercapacitor and Photo-Current Performance by in Situ Growth of AgNPs. *Colloids Surfaces A Physicochem. Eng. Asp.* **2024**, *685*, 133193. https://doi.org/10.1016/j.colsurfa.2024.133193.

(2) Nguyen, N. T.; Le, P. A.; Phung, V. B. T. Biomass-Derived Carbon Hooks on Ni Foam with Free Binder for High Performance Supercapacitor Electrode. *Chem. Eng. Sci.* **2021**, *229*. https://doi.org/10.1016/j.ces.2020.116053.

(3) Xu, X.; Sielicki, K.; Min, J.; Li, J.; Hao, C.; Wen, X.; Chen, X.; Mijowska, E. One-Step Converting Biowaste Wolfberry Fruits into Hierarchical Porous Carbon and Its Application for High-Performance Supercapacitors. *Renew. Energy* **2022**, *185*, 187–195. https://doi.org/10.1016/j.renene.2021.12.040.

(4) Zhang, D.; Sun, L.; Liu, Q.; Sun, H.; Wang, Q.; Li, W.; Li, Z.; Wang, B. Ultra-High Specific Surface Area Porous Carbon Derived from Chestnut for High-Performance Supercapacitor. *Biomass and Bioenergy* **2021**, *153* (July), 106227. https://doi.org/10.1016/j.biombioe.2021.106227.

(5) Yue, X.; Yang, H.; Cao, Y.; Jiang, L.; Li, H.; Shi, F.; Liu, J. Nitrogen-Doped Cornstalk-Based Biomass Porous Carbon with Uniform Hierarchical Pores for High-Performance Symmetric Supercapacitors. *J. Mater. Sci.* **2022**, *57* (5), 3645–3661. https://doi.org/10.1007/s10853-022-06891-9.

(6) Chen, W.; Wang, X.; Liu, C.; Luo, M.; Yang, P.; Zhou, X. Rapid Single-Step Synthesis of Porous Carbon from an Agricultural Waste for Energy Storage Application. *Waste Manag.* **2020**, *102*, 330–339. https://doi.org/10.1016/j.wasman.2019.10.058.

(7) Thirumal, V.; Yuvakkumar, R.; Ravi, G.; Dineshkumar, G.; Ganesan, M.; Alotaibi, S. H.; Velauthapillai, D. Characterization of Activated Biomass Carbon from Tea Leaf for Supercapacitor Applications. *Chemosphere* **2022**, *291* (P2), 132931. https://doi.org/10.1016/j.chemosphere.2021.132931.

(8) Zhang, J.; Chen, H.; Ma, Z.; Li, H.; Dong, Y.; Yang, H.; Yang, L.; Bai, L.; Wei, D.; Wang, W. A Lignin Dissolution-Precipitation Strategy for Porous Biomass Carbon Materials Derived from Cherry Stones with Excellent Capacitance. *J. Alloys Compd.* **2020**, *832*, 155029. https://doi.org/10.1016/j.jallcom.2020.155029.

(9) Xu, M.; Huang, Q.; Lu, J.; Niu, J. Green Synthesis of High-Performance Supercapacitor Electrode Materials from Agricultural Corncob Waste by Mild Potassium Hydroxide Soaking and a One-Step Carbonization. *Ind. Crops Prod.* **2021**, *161* (July 2020), 113215. https://doi.org/10.1016/j.indcrop.2020.113215.

(10) Baig, M. M.; Gul, I. H. Conversion of Wheat Husk to High Surface Area Activated Carbon for Energy Storage in High-Performance Supercapacitors. *Biomass and Bioenergy* **2021**, *144* (July 2020), 105909. https://doi.org/10.1016/j.biombioe.2020.105909.

(11) Charoensook, K.; Huang, C.-L.; Tai, H.-C.; Lanjapalli, V. V. K.; Chiang, L.-M.; Hosseini, S.; Lin, Y.-T.; Li, Y.-Y. Preparation of Porous Nitrogen-Doped Activated Carbon Derived from Rice Straw for High-Performance Supercapacitor Application. *J. Taiwan Inst. Chem. Eng.* **2021**, *120*, 246–256. https://doi.org/10.1016/j.jtice.2021.02.021.

(12) Li, S.; Chen, Q.; Gong, Y.; Wang, H.; Li, D.; Zhang, Y.; Fu, Q.; Pan, C. "One-Step" Carbonization Activation of Garlic Seeds for Honeycomb-like Hierarchical Porous Carbon and Its High Supercapacitor Properties. *ACS Omega* **2020**, *5* (46), 29913–29921. https://doi.org/10.1021/acsomega.0c04190.

(13) Phiri, J.; Dou, J.; Vuorinen, T.; Gane, P. A. C.; Maloney, T. C. Highly Porous Willow Wood-Derived Activated Carbon for High-Performance Supercapacitor Electrodes. *ACS Omega* **2019**, *4* (19), 18108–18117. https://doi.org/10.1021/acsomega.9b01977.

(14) Yu, P.; Liang, Y.; Dong, H.; Hu, H.; Liu, S.; Peng, L.; Zheng, M.; Xiao, Y.; Liu, Y. Rational Synthesis of Highly Porous Carbon from Waste Bagasse for Advanced Supercapacitor Application. *ACS Sustain. Chem. Eng.* **2018**, *6* (11), 15325–15332. https://doi.org/10.1021/acssuschemeng.8b03763.

(15) Chen, X.; Chi, M.; Xing, L.; Xie, X.; Liu, S.; Liang, Y.; Zheng, M.; Hu, H.; Dong, H.; Liu, Y.; Jiang, S. P.; Xiao, Y. Natural Plant Template-Derived Cellular Framework Porous Carbon as a High-Rate and Long-Life Electrode Material for Energy Storage. *ACS Sustain. Chem. Eng.* **2019**, *7* (6), 5845–5855. https://doi.org/10.1021/acssuschemeng.8b05777.

(16) Guan, L.; Pan, L.; Peng, T.; Gao, C.; Zhao, W.; Yang, Z.; Hu, H.; Wu, M. Synthesis of Biomass-Derived Nitrogen-Doped Porous Carbon Nanosheests for High-Performance Supercapacitors. *ACS Sustain. Chem. Eng.* **2019**, *7* (9), 8405–8412. https://doi.org/10.1021/acssuschemeng.9b00050.

(17) Peng, L.; Liang, Y.; Huang, J.; Xing, L.; Hu, H.; Xiao, Y.; Dong, H.; Liu, Y.; Zheng, M. Mixed-Biomass Wastes Derived Hierarchically Porous Carbons for High-Performance Electrochemical Energy Storage. *ACS Sustain. Chem. Eng.* **2019**, *7* (12), 10393–10402. https://doi.org/10.1021/acssuschemeng.9b00477.

(18) Chen, F.; Ji, Y.; Deng, Y.; Ren, F.; Tan, S.; Wang, Z. Ultrasonic-Assisted Fabrication of Porous Carbon Materials Derived from Agricultural Waste for Solid-State Supercapacitors. *J. Mater. Sci.* **2020**, *55* (25), 11512–11523. https://doi.org/10.1007/s10853-020-04751-y.

(19) Yin, Z.; Xu, Y.; Wu, J.; Huang, J. Effect of Pomelo Seed-Derived Carbon on the

Performance of Supercapacitors. *Nanoscale Adv.* **2021**, *3* (7), 2007–2016.

https://doi.org/10.1039/d0na00778a.
